# **Using *Buzzy*** ^*®*^ in venipunctures: perceptions of children and adolescents with congenital cardiopathies^*^

**DOI:** 10.1590/1518-8345.7822.4849

**Published:** 2026-08-10

**Authors:** Lívia Grazielle Benevides dos Santos, Michelle Darezzo Rodrigues Nunes, Lucila Castanheira Nascimento, Liliane Faria da Silva, Fernanda Machado Silva-Rodrigues, Bárbara Bertolossi Marta de Araújo

**Affiliations:** 1 Hospital Federal de Bonsucesso, Rio de Janeiro, RJ, Brazil.; 2 Universidade do Estado do Rio de Janeiro, Faculdade de Enfermagem, Departamento Materno-Infantil, Rio de Janeiro, RJ, Brazil.; 3 Scholarship holder of the Programa de Incentivo à Produção Científica, Técnica e Artística da Universidade do Estado do Rio de Janeiro, Rio de Janeiro, RJ, Brazil.; 4 Universidade de São Paulo, Escola de Enfermagem de Ribeirão Preto, PAHO/WHO Collaborating Centre for Nursing Research Development, Ribeirão Preto, SP, Brazil.; 5 Universidade Federal Fluminense, Escola de Enfermagem Aurora de Afonso Costa, Departamento Materno-Infantil, Niterói, RJ, Brazil.; 6 Universidade de São Paulo, Escola de Enfermagem, São Paulo, SP, Brazil.

**Keywords:** Child, Adolescent, Congenital Heart Defects, Pain, Pain Management, Pediatric Nursing.

## Abstract

**Objective:**

to understand the experiences undergone by children and adolescents with congenital cardiopathies in relation to using vibration along with cryotherapy (*Buzzy* ^*®*^ ) during venipunctures.

**Method:**

a qualitative, descriptive and exploratory study conducted with children and adolescents hospitalized due to congenital cardiopathies. The data were collected by means of audio-recorded semi-structured interviews, after using *Buzzy* ^*®*^ during venipunctures. The data were analyzed via inductive content analysis, as proposed by Elo and Kyngas.

**Results:**

the participants were 15 children and adolescents with a mean age of 11.1±3.6 years old. Four categories were created, which evidenced the experiences undergone by children and adolescents with cardiopathies when using *Buzzy* ^*®*^ , namely: Perceiving pain reduction or not; Experiencing physical sensations of relaxation and anesthesia; Getting distracted with *Buzzy* ^*®*^ ; and Reducing anxiety. It was identified that the participants experienced pain and anxiety reductions, anesthesia, relaxation and distraction when using the device, as only two perceived no difference.

**Conclusion:**

*Buzzy* ^*®*^ can be an effective non-pharmacology strategy to manage pain and anxiety during venipunctures in pediatric patients, as it showed benefits in the physical and emotional aspects, with the possibility of being using both in the clinical practice and in future research studies.

## Highlights

**(1)** The perceptions regarding how *Buzzy* ^*®*^ is used suggest pain reduction during venipunctures. **(2)** The participants’ experiences also show decreased anxiety. **(3)** The importance of using non-pharmacological strategies in procedures is evidenced.

## Introduction

Congenital heart diseases constitute one of the most prevalent chronic conditions in children and adolescents and, as well as in several childhood chronic pathologies, they require frequent visits to hospitals, with the possibility of prolonged hospitalizations and physical and emotional wear out due to routine physical examinations pre-established in therapeutic plans. Staying in a hospital environment is stressful, as children undergo treatments, changes in their routine and multiple painful procedures^1^.

By itself, pain is a traumatic event for children, as it causes intense physical discomfort and repercussions in their emotional, psychological and behavioral aspects. When faced with pain, they frequently indulge in fantasy thoughts characteristic of the development phase they are undergoing, where pain and fatality can be considered synonyms^2^.

Among the set of invasive procedures performed with needles, venipunctures are one of the main causes of pain and distress in pediatric patients. These acute nociceptive experiences can trigger undesirable psychological, emotional and behavioral repercussions in terms of pain perception^3^.

Such sensation is directly associated with consequences for children with cardiac malformations, including severe cyanosis with tissue hypoxia, reduced cardiac debit and nutritional alterations. These effects are the result of the reduction in pulmonary blood flow and of the increase in stress-triggering hormones^4^.

Most of the times, the first alternative to reduce pain and its consequences during routine painful procedures performed in children with cardiopathies is to use drugs such as opioids and analgesics^5^. However, a number of studies show that the metabolism of medications is directly influenced by the cardiac status of children with heart diseases; therefore, perfusion alterations can impair drug metabolization and increase the poisoning risk, as well as cause hemodynamic and respiratory side effects capable of further worsening health conditions^5^.

Therefore, one of the alternatives in the assistance provided to children and adolescents in pain is to use non-pharmacological methods; one of them is *Buzzy* ^*®*^ , a plastic portable technological device in the form of a bee, ladybug or butterfly, which combines cold from its freezable wings (cryotherapy) with the vibratory stimuli from the instrument›s body. Such device has shown its ability to reduce fear, distress and anxiety in children, in addition to providing peace of mind to parents and benefiting health professionals^6^. The effect exerted by *Buzzy* ^*®*^ is based on the “Gate Control Theory” discovered by Melzack and Wall in 1965, which states that it is possible to control the synaptic information of pain by activating nociceptive fibers. This mechanism blocks the transmission of painful sensations in the different fibers receptive to pain (A-Delta and C fibers) by means of the cold and vibrations emitted by the device^7^.

Based on the Symptom Management Theory (SMT)^8^, the theoretical framework guiding the study, it is deemed necessary to maintain pain management and control even when it is not a symptom but a consequence of an invasive procedure. Consequently, the theory can assist in identifying pain experiences and in the strategies to minimize them.

However, although various randomized clinical trials have shown the efficacy of *Buzzy* ^*®(*^ ^6^, the literature still lacks qualitative studies researching the children’s perception as for using this device during procedures involving needles^9^. Thus, understanding that the children’s and adolescents’ opinion is essential to propose any type of intervention that is performed on them and acknowledging them as moral agents, subjects with rights and active participants in their health care process^10^, this study aimed at understanding the experiences undergone by children and adolescents with congenital cardiopathies in relation to using *Buzzy* ^*®*^ during venipunctures.

## Method

### Type of study

A qualitative, descriptive and exploratory study. The research was prepared and presented according to the Consolidated Criteria for Reporting Qualitative Research(COREQ).

### Data collection *locus* and period

This study was conducted at the Cardiopediatrics ward of a federal hospital that is a reference in the treatment of complex heart diseases in Rio de Janeiro, Brazil.

Data collection took place between September 2023 and March 2024 until reaching data saturation; in other words, when no new or relevant information was obtained to develop the categories and, therefore, the data collected were deemed sufficient to understand the phenomenon researched^11^.

### Participants and selection criteria

The study participants were 15 pediatric patients that met the following inclusion criteria: children and adolescents with congenital cardiopathies aged between six and 18 incomplete years old, of both genders, hospitalized in the data collection *locus*, subjected to venipuncture procedures and not presenting any cognitive impairment.

Venipunctures should be understood as procedures in which a needle is used to penetrate the skin and the blood vessels, in order to allow collecting blood samples and performing intravenous treatments through peripheral venous accesses^12^.

The children and adolescents excluded from the study were those in Palliative Care, those in critical hospitalization situations and those that had been administered any type of sedative or analgesic to perform the procedures, given the possibility of biases in the results. Children and adolescents that still lacked a defined diagnosis and those who were in contact, droplet or aerosol precautions were also excluded due to the cross-contamination risk if *Buzzy* ^*®*^ was used.

The children and adolescents that met the aforementioned criteria were invited to participate in person and there was no refusal or impossibility to take part in the research.

### Data collection procedures

The characterization data corresponding to each child and person responsible for them (birth date, gender, treatment undergone and hospitalization reason, for example) were collected directly during the interviews or by consulting the patients’ medical records.

After agreeing to participate and once the person responsible for each patient had signed a Free and Informed Consent Form and the children/adolescents had signed an Assent Form, the procedures for using *Buzzy* ^*®*^ XL (Extra Large) in the form of bee during the venipunctures were initiated. At a first moment, the nurse working in the sector and in charge of the procedure inspected each child’s or adolescent’s upper limbs to choose the vein and the respective puncture site.

Subsequently, approximately one minute before the puncture, the researcher attached the bee’s silicone wings that had been stored in the Cardiopediatrics ward refrigerator to the vibratory body of the *Buzzy* ^*®*^ device. The device was then positioned over the needle entry point and fixed with a silicone band that comes with the device, so that the professional had both hands free for the procedure. Once positioned and fixed, the device was turned on for 30 to 60 seconds. At the puncture moment, the device was moved to approximately five centimeters above the puncture site and kept on for at least another 60 seconds once the procedure had ended.

In order to ensure rigor in the way the research was developed, the nurses from the Cardiopediatrics sector that performed the venipunctures were previously instructed about the device and its function and undergone due training about how to apply it and the step-by step procedure to be followed during data collection. So as to avoid implications in the results, aspects such as application time over the needle entry point, distance to move the device in relation to the needle entry point and time for the cold component to remain outside the refrigerator were standardized across the professionals. The lead researcher (LGBS) was present during the venipunctures in all data collection instances, assisting the nurses in applying the device.

In the cases where a second attempt was necessary to find the peripheral access (P1, P9 and P14), *Buzzy* ^*®*^ was kept over the first attempt site until the nurse identified the new puncture site. Subsequently, the same procedures for using *Buzzy* ^*®*^ XL were followed in the new site.

In some cases, it was necessary to wait some minutes after the procedure for the children or adolescents to have the physical sensations inherent to the venipuncture attenuated. After this period, the interviews were initiated and each child or adolescent was invited to answer some questions, such as the following ones: “What was it like for you to use *Buzzy* ^*®*^ during the procedure?”, “If 0 represents “No pain” and 10 is “The maximum pain possible”, how much pain did you feel when using the little bee, from 0 to 10?” and “Have you already been punctured before? How much pain did you feel in that previous experience, from 0 to 10?”. As each question was answered, others were asked. Phrases such as *“Can you explain?”* and *“Tell me more about this”* sought to deepen on the answers found. All the children and adolescents were previously instructed about the numerical scale for pain.

The *Buzzy* ^*®*^ device was disinfected before and after each use by rubbing it with gauze moistened with a 70% alcohol solution. The interviews were audio-recorded with a cell phone and conducted slowly, so as to detect if any discomfort or negative emotion arose in the participants, not identifying any. They were subsequently transcribed in full to read and analyze them.

### Data analysis

The qualitative data obtained from the semi-structured interviews were transcribed and the content was analyzed according to the inductive content analysis stages, as per the following steps: Preparation, Organization and Reporting of the results^13^.

The Preparation phase was initiated by transcribing the answers to the semi-structured interviews. There are no systematic rules for data analysis in this stage, as the words from the text are grouped into smaller content categories. To such end, the materials were thoughtfully read in order to identify the meanings contained in the participants’ answers and disentangle them. In the Organization phase, the data were openly coded, grouped and synthesized based on their similarities and differences. Finally, the categories were created with no restrictions. The results were reported by naming the categories and subcategories, according to the characteristics of the content analyzed^13^.

In relation to the sample characterization data, the nominal dependent variables (gender, diagnosis, skin color, kinship, schooling, marital status, hospitalization reason and origin) were analyzed and described in terms of distribution of absolute (n) and relative (%) variables; in turn, the continuous dependent variables (age and income) were reported by analyzing central tendency (mean) and dispersion (standard deviation) measures^14^.

### Ethical aspects

The project was submitted to and approved by the Ethics Committees for Research (*Comitês de* Ética *e Pesquisa*, CEPs) with Human Beings of the proposing and co-participating institutions, by means of Process No. 6,132,990 and CAAE No. 68806623.9.0000.5282. All the stages observed the guidelines set forth in National Health Council Resolution No. 466/2012^15^.

In the data collection procedures and after identifying the participants that met the selection criteria, those responsible for the patients were presented the research topic and objectives. After agreeing to participate, each person responsible signed a Free and Informed Consent Form and each children/adolescent signed an Assent Form. All the information obtained in this study was treated in a confidential way, with secrecy guaranteed by resorting to coding, where “P” means “Participant” and the respective numbers from 1 to 15 correspond to the order in which the interviews were conducted, followed by each participant’s age.

The participants were provided with guidelines about their rights, the research objectives and minimum risk of emotional discomfort during the interview, given the moment marked by frailty they were experiencing during their hospitalization. They were also advised about the possibility of withdrawing from the study at any moment and with no harm.

It is stated that there are no conflicts of interests in using the *Buzzy* ^*®*^ System in this research.

## Results

The study participants were 15 children and adolescents with congenital cardiopathies. Their mean age was 11.1±3.6 years old, with seven (n=7) children aged between 11 (47%) and eight (n=8) adolescents aged from 12 to 17 (53%). Most of them were male (n=8, 53%) and were hospitalized for surgical treatments (n=13, 86%). As for their diagnoses, acyanotic cardiopathies prevailed (n=6, 40%).

All the participants were punctured with flexible catheters measuring 22 or 24 in diameter, according to the evaluation made by the nurse in charge of the procedure. The punctures were performed in the upper limbs: antecubital fossa (n= 4), back of the hand (n=7) or forearm (n=4). Only in three (n=3) punctures was it necessary to make two attempts to find the access.

Of all 15 participants, 13 mentioned less pain when using *Buzzy* ^*®*^ and two assessed puncture pain as with the same intensity when using the device.

After analyzing the interviews, four categories and one subcategory were created, as presented below.

### Perceiving pain reductions or not

When invited to answer questions about their pain experience when using *Buzzy* ^*®*^ , most of the participants stated that they did not at all feel the needle enter their body at the puncture moment thanks to the device, thus considering that pain was alleviated. According to them, that contributed for the venipuncture experience to present less discomfort, with positive consequences for venipunctures in general, for avoiding crying and abrupt limb movements at the time of the procedure. In addition to that, they were delighted with the decreased sensitivity and the consequent reduction in the painful sensation.

*The little bee doesn’t hurt. I think that I’m not going to cry any more [...] I liked her very much, I’m going to kiss her! (P1, 9 years old).*

*[...] In my own experience, right?, I felt the needle entering, but I didn’t feel the bite thanks to the vibration [...] It helps a lot that you also not feel the needle and then you end up delighted (P4, 13 years old).*

*Because it left the arm almost lifeless, it didn’t burn, the cold helps with the vibration, you still feel it, but just a little. There’s almost no pain (P13, 13 years old).*

*It was different, I didn’t even retract my hand when she (nurse) applied it, because I didn’t even feet the needle, it was quick and easy [...] I didn’t feel much discomfort. I did feel the little device, right?, it was good because it’s as if pain was less (P15, 12 years old).*

For the participants, *Buzzy* ^*®*^ was able to significantly reduce the painful bite sensation at the time of the venipuncture, when compared to the procedures with needles they had undergone before the research without using the device.

*Researcher: From 0 to 10, how would you rate your pain in those other experiences? Adolescent: I think that it was eight or nine. Researcher: And now, after using the little bee, how do you rate your pain from 0 to 10? Adolescent: Three (P4, 13 years old).*

*Researcher: And from 0 to 10, how do you rate your pain in those other injections you’ve already had? 0 means “No pain” and 10 is “A lot of pain”. Adolescent: It hurt a lot. An eight, let’s say. Researcher: And when using the little bee, from 0 to 10, how much did the injection hurt? Adolescent: Only two (P6, 15 years old).*

*Researcher: If you had to rate with 0 for “It doesn’t burn at all”, 1 for “it burns a little”, 2 “It burns a little more”, 3 “It burns more already”... until reaching 10 for “It burns a lot”, which number would you choose? Child: Ten. Researcher: Understood, and with the little bee today? Which number? Child: One (1) (P11, 8 years old).*

Two (n=2) participants mentioned equivalent experiences in relation to pain intensity with and without the little bee; they were the same ones that required two punctures for the procedure to be successful. According to them, when using the device during the venipuncture, pain was quite similar to the one felt in previous experiences.

*Researcher: Then, from 0 to 10, how much pain did you feel using the little bee? Adolescent: Well, I’m going to rate it the same as with normal blood draws: 5 or 6, let’s say (P9, 13 years old).*

*Researcher: And how would you rate, from 0 to 10, the pain you felt when the needle got in and you were with the little be? Child: I say 7. Researcher: And without the little bee those other times? Child: Also 7. It was very similar, it hurt the same (P14, 9 years old).*

### **Highlighting improvement possibilities in using *Buzzy*** ^*®*^

Some of the participants’ perceptions about using the device assisted in recognizing some limitations and improvement suggestions to use this tool, in order to enhance pain reduction. Two participants (P5 and P9) noted that the cold component might be more effective if its freezing time was longer and another one (n=1) stated that the vibration was low intensity and, therefore, that it was scarcely perceived. It is highlighted that these two participants were the same ones that required more than one puncture for the procedure to be successful, which demanded more time to perform the procedure.

*If she was a little colder, I think that she’d anesthetize better. I didn’t feel the vibration. [...] I only felt her wings (P9, 13 years old).*

*[...] If the little bee was a little colder... because it was somewhat liquid, right? (P5, 11 years old).*

### Experiencing physical sensations of relaxation and anesthesia

Through the participants’ testimonies, it was observed that *Buzzy* ^*®*^ can provide relaxation and anesthesia sensations. For them, the ice and vibration in contact with the skin trigger sensations of muscle relaxation, numbness, tingling and anesthesia in the punctured limb. For this reason, they feel that the puncture site is massaged, paralyzed or even numbed and lifeless. As a consequence, their puncture site becomes less sensitive during the procedure, which helps them face that extremely feared puncture moment.

*It’s good. [...] Because it’s relaxing. [...] I feel some relaxation that’s good (P7, 9 years old).*

*It was vibrating, right? It was in contact there, it relaxed me a little (...) The arm, you know? Then I felt as if my arm was a little anesthetized, but only the arm. The anesthesia is as if you feel the arm a little numb and you don’t feel pain in the area. Because the ice anesthetizes when you put it over something. As it anesthetized here (points to the site where Buzzy* ^*®*^ was placed) (P9, 13 years old).

*She made me feel no pain, I felt some tingling and that’s all (...) It didn’t hurt at all, because of that tingling and trembling sensation, it makes me not feel the normal needle (P10, 6 years old).*

*When she placed the ice, it went numb to place the needle and it didn’t even look as if it had a needle. It’s because the ice left my hand numb, and I didn’t even feel the needle much, I only felt a really mild bite (P12, 13 years old).*

*Because she acts differently, my arm tingles and is somewhat paralyzed. The sensation is that it’s less sensitive (P14, 9 years old).*

### **Getting distracted with *Buzzy*** ^*®*^

The children and adolescents also made comments as for the aspect of *Buzzy* ^*®*^ , using words such as pretty, interesting, similar to a toy, merry and happy. These adjectives were related to distraction when the needle procedures were performed, which are usually stressful. The wish to interact with the device itself was also identified in the reports.

They assessed the instrument appearance as an adjuvant to provide peace of mind, distraction and happiness; they also related the bee shape to familiarity with the drawings they found in their everyday life. For this population group, all these characteristics conferred friendliness to the device, helped entertain them and made them shift their focus away from the venipuncture.

*She’s cute. I found it very interesting because its aspect is certainly going to calm the children, they’re going to find it pretty, it’s going to be a distraction. It’s going to make the children not think about the needle, but in the little bee. (P2, 14 years old).*

*Because it’s pretty, her little face, it’s cool [...] she’s smiling, she’s happy (P14, 9 years old).*

*It’s just like a toy (...) Because of the noise. Because of the wings too! It’s also good because it has her shape. It has those drawings. A smile in her face. [...] It’s just like the drawings I make at school, but I can’t draw the little face like that. (...) Happy and merry. It makes her prettier and makes you want to grab her. [...] she distracts me. [...] she helps me not think about the needle (P10, 6 years old).*

Despite that, the same child from the previous testimony stated as a counterargument the possibility that the aesthetics of *Buzzy*D ^*®*^ might trigger contradictory feelings, as bee bites can be painful.

*Child: When it vibrated, I thought that the little bee was with the needle, but then I saw it wasn’t and that it stopped hurting. Researcher: And why did you think that she had a needle? Child: Because bees have that thing that bites (P10, 6 years old).*

### Reducing anxiety

Through the reports it was observed that *Buzzy* ^*®*^ reduces venipuncture-associated anxiety for two reasons: children and adolescents place expectations regarding pain reduction when using the device and the very sensory resources of the device (vibration and cold) favor analgesia. Thus, when shifting their attention to the device and perceiving the reduction in the painful sensation, they stopped thinking about the puncture and became calmer and more relaxed. In their experiences, fear of the needle, nervousness and fright linked to the procedure were minimized when using *Buzzy* ^*®*^ .

For resulting in reduced tension at a moment marked by vulnerability, they praise application of the device as a very good intervention, delightful and with the potential to soothe them.

*[...] The fact of me imagining that it’s not going to hurt so much also calms you down a lot, it removes that nervousness that normally disturbs you at that moment (P2, 14 years old).*

*I was just delighted! I think that for anyone who’s really afraid of needles, as in my case, for anyone with some fear or who’s afraid of blood, it’s also very good for that time (P4, 13 years old).*

*When you placed the little bee and left it vibrating, it was already better because it already soothed me [...] she calms you down because she makes you not feel so much pain. I’ve always been afraid of needles, but... there was no other way, and it looked as if I was pinching into infinity until they removed the needle. And, with the little be, I felt a whole lot better (P12, 13 years old).*

*Because it hurt less at the time, less fear. When the needle got it, kinda I didn’t feel so much as before, I didn’t have that fear reaction then [...] it had to be that from the other times to help reduce that fear (P15, 12 years old).*

## Discussion

The participants’ subjective perceptions and experiences indicate that the *Buzzy* ^*®*^ device reduces pain and anxiety during procedures with needles in children and adolescents with congenital cardiopathies because it generates anesthesia and tingling sensations, promotes muscle relaxation and favors distraction.

Despite the usual need for venipunctures in hospitalization units to perform tests and the treatments proposed, they are pain-triggering procedures and, because of that, they cause fear, anxiety and physical and emotional stress^16^. All these feelings and stressful agents experienced continuously can contribute to impairments in children’s and adolescents’ growth and development, as they affect the central nervous system, cognition, and motor and emotional improvements. In addition, they can harm children’s and adolescents’ adherence to health care in the future, which confers utmost importance to this theme^17^.

Despite the many advances in pain evaluation and treatment in the pediatric population, there are still difficulties both quantifying it due to its subjective nature and treating it properly. As a consequence, children and adolescents with cardiopathies experience emotional and sensory distress, with a risk of undergoing important hemodynamic alterations, with tissue oxygenation variations due to intravascular pressure^18^. Therefore, it is necessary to find a way to render the physical and emotional experience more pleasant even during procedures with needles, in order to humanize hospitalization and minimize the negative impacts on quality of life^19^.

A recent literature review^18^ investigated the main non-pharmacological interventions for pain management in children aged over one year old subjected to procedures with needles and concluded that the three main methods adopted for peripheral venipunctures were chewing gum, distraction and *Buzzy* ^*®*^ .

Based on the Symptom Management Theory (SMT), the theoretical framework that guided this study^7^, it is understood that the responses to symptoms such as pain can encompass physiological, psychological and behavioral dimensions and that the best way to comprehend that experience is through self-reports by those who undergo them. Therefore, it was sought to develop a more precise and deeper approach toward this phenomenon from the qualitative point of view, based on the perspective of children and adolescents with congenital cardiopathies, with the intention of exploring the meanings they attribute to the evaluations about using this device and the ways in which they respond to the symptom after applying the intervention proposed.

In the reports, it was verified that the intervention resulted in reduced pain for almost all the participants, except two of them, who perceived no change in their pain level. This can be explained by the existing relationship between the mechanisms for the local analgesia effect generated during punctures and by the shared synapses in the spine, where the fibers that transport painful stimuli share pathways with those in charge of thermal and mechanical conduction. This interneural interference in synaptic conduction can generate the relaxation sensation mentioned by the participants and, consequently, pain relief^20^.

Pain modulation with *Buzzy* ^*®*^ takes place due to the influence exerted by this technological device on the descending inhibitory system, which originates in the mesencephalon gray substance. In this case, the pain-related information that would leave the encephalon toward the spine dorsal horn is reduced as a result of serotoninergic alterations^21^. As a consequence, physical pain and emotional distress are mitigated when using *Buzzy* ^*®*^ .

In addition to the benefits for the patients, the procedure becomes less traumatic for the professionals in charge of performing the punctures, as they can be made faster and easily, with fewer chances of the patients crying, getting agitated and moving abruptly. Therefore, using *Buzzy* ^*®*^ can render the procedures safer and increase the professionals’ success chances in the first puncture attempt. Several sensations attributed to the device such as “not crying”, “calms you down”, “not feeling so nervous” and more “relaxed” assist in this process.

Additionally, pain reduction comes along “anesthesia”, “relaxation”, “numbness” or “sleepiness”, “paralysis” and “tingling” sensations in the reports. Combining cryotherapy with vibration has its efficacy also enhanced by the release of endogenous opioids resulting from the activation of cold-related touch receptors, which causes a local numbness sensation with consequent “anesthesia”, according to the participants’ reports^21^.

A clinical study prepared by the woman who developed the technology in question compared different frequency vibrations to determine which one would be the best to be applied in the device. It was verified that the 180-200 Hz frequency applied to the Oscillice technology of *Buzzy* ^*®*^ is powerful enough to outdo the effects of therapies based on electrical stimulation in reducing post-surgical and spine pain, among other lesions^22^. In agreement with these results, another study showed that high-frequency mechanical vibration significantly relieves musculoskeletal pain, proving to be more effective than traditional methods (even pharmacological ones), which corroborates the reports by the participants included in this study^23^.

Also regarding the sensations mentioned by the children and adolescents, it becomes important to note that the local cooling effect exerted by the ice wings provides analgesia and contributes to numbness by reducing the local metabolic rate and decreasing the flow of inflammatory mediators. Interrupting the transmission of pain signals in this way successively increases the absolute pain threshold with increased beta endorphins; in addition, the muscles become “relaxed” and “numb” in a short time, easing the procedure with needles^24^. Beyond the elements previously described about the device that generate analgesia, distraction is one of the mechanisms that confer effectiveness to *Buzzy* ^*®*^ and has been tested in various randomized clinical trials^21^.

The playful trait of the device remits children to game settings and is closely linked to emotional and cognitive development. According to a study, when professionals use playful elements properly through participatory care, they can help children structure the events around them, for reducing anxiety, increasing treatment acceptance, improving communication and assisting in attributing new meanings to the hospitalization experience^25^.

In international articles it was identified that, when nervous in procedures with needles, children and adolescents can present a considerable increase in their heart rate^21^ ^,^ ^26^ ^-^ ^28^, in addition to hypoxemia and vasovagal and hormonal changes^21^ ^,^ ^28^. When using *Buzzy* ^*®*^ , a reduction toward levels close to normality was noticed in the cardiac parameters^26^ ^-^ ^27^.

Although children and adolescents acknowledge *Buzzy* ^*®*^ as a useful instrument to reduce procedure-related pain, the findings reveal aspects inherent to the device that represent challenges and improvement possibilities. In this sense, it is considered important to devise more adaptive protocols that take into account developmental, sensory and contextual variables, in order to optimize these individuals’ experiences^26^.

In this study, two participants mentioned no relief in their pain sensation. Although most studies show the efficacy of the device, other surveys corroborate the need for individualized strategies^29^. It is noted that both participants are the same ones for whom the first attempt to find the peripheral access was unsuccessful. In addition to that, one of them mentioned an improvement opportunity in relation to the freezing time for the wings. According to a study^25^, repeated peripheral catheterization attempts can increase children’s and adolescents’ sensitivity to this type of procedure and potentate pain, as procedures with needles are the first cause of fear among these patients^25^.

Another participant that needed a second puncture mentioned a reduction in the pain sensation during the procedure. Thus, it is believed that various factors may influence pain sensations, such as each child’s developmental stage^7^ ^,^ ^28^, greater difficulty for pain modulation in children aged less than seven years old, variations in the way the technique is applied, cryotherapy exposure time, individual preferences in terms of colors and shapes that may not be in line with the device presentation^24^ ^,^ ^26^ and presence of the parents^30^.

Contradictorily to the playful perspective of the device, there was an association between the bee format of *Buzzy* ^*®*^ and imminent risks for painful sensations related to the insect biting. Younger children incorporate fantasy elements into their realities^20^, which may render the little bee frightening for some of them. In this case, choosing the ladybug variation might be an option.

In addition, two participants considered that the freezing time for the little bee wings was insufficient to provide adequate analgesia, as they were already in liquid state during the punctures. It is known that the defreezing time for the cold component from the moment that it is removed from a freezer is 10 minutes^23^. This means that, for longer procedures or those requiring more time for their operationalization, it would be important to remove the little wings from the freezer as close as possible to the beginning or to replace it by another one.

As study limitations, we can note the need for a *Buzzy* ^*®*^ format that is more suitable for the users’ age group, as well as to replace the frozen silicone wings when they are out of the refrigerator for more than 10 minutes.

Conferring voice to children and adolescents is an important strategy for their empowerment and inclusion in the care process, as it eases their approach to health teams and to scientific knowledge. In parallel, a number of studies reinforce that children’s and adolescents’ self-reports about their health status or experiences is the best way to understand their opinions, turning each testimony into an extremely valuable element to comprehend the whole^8^ ^,^ ^10^ ^,^ ^25^.

The following is noted as a strength of this study: the encouragement for health teams to use non-pharmacological interventions, an option still scarcely chosen in the clinical practice. For example, this can be noticed in a study that evaluated 1,251 medical records corresponding to hospitalized children and identified Nursing notes about the use of non-pharmacological measures for pain management such as local heat, distraction and cold compresses^2^ only in six of them. It is understood that, as it is comprised by three components (the symptom experience: pain in this study; the strategies for pain management: *Buzzy* ^*®*^ ; and the results: pain when using *Buzzy* ^*®*^ ), the SMT has the potential to exert an influence on institutional practices and to devise suitable care environments to enhance quality of life in this population group, in addition to guiding Nursing interventions with a view to proper symptom management in the practice^8^.

New research studies about using *Buzzy* ^*®*^ in children and adolescents subjected to painful procedures and those where needles are employed should be conducted to deepen on these findings and investigate the experiences undergone by this pediatric population in a broader variety of procedures, treatment situations (inside and outside hospitals) and diagnoses, and with different designs (such as randomized clinical studies with mixed approaches). Consequently, it will be possible to deepen on what is known about the effects of*Buzzy* ^*®*^ .

## Conclusion

The four categories found (“Perceiving pain reductions or not”, “Experiencing physical sensations of relaxation and anesthesia”, “Getting distracted with *Buzzy* ^*®*^ ” and “Reducing anxiety”) evidence the experiences undergone by children and adolescents with cardiopathies when using *Buzzy* ^*®*^ during venipunctures. In the participants’ assessments, this is an effective, interesting and delightful non-pharmacological intervention that acts on their physical and emotional dimensions alike, as they experience less pain, anxiety and nervousness during the procedures and stay more relaxed and distracted.

It is believed that the results obtained in this study can contribute to research, as they reduce the national and international gap evidenced in the literature search. They also assist in sensitizing other professionals about the importance of using pain reduction strategies during venipunctures, so as to qualify Nursing assistance for children and adolescents and rendering care humane and less traumatizing.

## Data Availability

Datasets related to this article will be available upon request to the corresponding author.

## References

[1] Spillmann R, Polentarutti S, Ehrler M, Kretschmar O, Wehrle FM, Latal B (2023). Congenital heart disease in school-aged children: cognition, education, and participation in leisure activities. Pediatr Res.

[2] Carvalho JA, Souza DM, Domingues F, Amatuzzi E, Pinto MCM, Rossato LM (2022). Pain management in hospitalized children: a cross-sectional study. Rev Esc Enferm USP.

[3] Oliveira NCAC, Gaspardo CM, Linhares MBM (2017). Pain and distress outcomes in infants and children: a systematic review. Braz J Med Biol Res.

[4] Amdani S, Conway J, George K, Martinez HR, Asante-Korang A, Goldberg CS (2024). Evaluation and management of chronic heart failure in children and adolescents, including those with congenital heart disease: a scientific statement from the American Heart Association. Circulation.

[5] Holladay J, Winch P, Morse J, Anderson BJ, McKee CT, Rice-Weimer J (2023). Acetaminophen pharmacokinetics in infants and children with congenital heart disease. Paediatr Anaesth.

[6] Jin F, Wang X, Qi M, Zhang W, Zhang Y (2024). Effectiveness and safety of Buzzy device in needle-related procedures for children under twelve years of age: a systematic review and meta-analysis. Medicine (Baltimore).

[7] Susam V, Friedel M, Basile P, Ferri P, Bonetti L (2018). Efficacy of the Buzzy system for pain relief during venipuncture in children: a randomized controlled trial. Acta Biomed.

[8] Humphreys J, Lee KA, Carrieri-Kohlman V, Puntillo K, Faucett J, Janson S, Smith MJ, Liehr PR (2008). Middle range theory for nursing.

[9] Santos LGB (2024). O uso da vibração associada à baixa temperatura no gerenciamento da dor durante punção venosa em crianças com cardiopatia congênita hospitalizadas.

[10] Cashmore J, Kong P, McLaine M (2023). Children’s participation in care and protection decision-making matters. Laws.

[11] Rahimi S, Khatooni M (2024). Saturation in qualitative research: an evolutionary concept analysis. Int J Nurs Stud Adv.

[12] Yamada Y, Kitamura M, Inayama E, Kishida M, Kataoka Y, Ikenoue T (2023). Acoustic stimulation for relieving pain during venipuncture: a systematic review and network meta-analysis. BMJ Open.

[13] Elo S, Kääriäinen M, Kanste O, Pölkki T, Utriainen K, Kyngäs H (2014). Qualitative content analysis: a focus on trustworthiness. SAGE Open.

[14] Dar NA, Tali TA, Ganie BA, Sofi MA, Sofi SR, Khan NA (2023). Statistical methods used in medical research and cancer registries: a review. J Radiat Cancer Res.

[15] Brasil (2013). Dispõe sobre diretrizes e normas regulamentadoras de pesquisas envolvendo seres humanos. Resolução nº 466, de 12 de dezembro de 2012.

[16] Cho YH, Chiang YC, Chu TL, Chang CY, Chang CC, Tsai HJ (2022). The effectiveness of the Buzzy device for pain relief in children during intravenous injection: quasi-randomized study. JMIR Pediatr Parent.

[17] Suleman SK, Atrushi A, Enskär K (2022). Effectiveness of art-based distraction in reducing pain and anxiety of hospitalized children during cannulation procedure: a randomized controlled trial. Belitung Nurs J.

[18] Mendes BV, Furlan MS, Sanches MB (2022). Non-pharmacological interventions in painful needle procedures in children: integrative review. BrJP.

[19] Fuchs A, Cordes BL, Van Dick R, Ebers G, Kaluza A, Konietzny C (2024). Interventions to alleviate anxiety and pain during venipuncture in children with chronic gastrointestinal and/or liver disease: a single-center prospective observational study. JPGN Rep.

[20] Narimany R, Faghihian R, Samani MJ (2024). Effectiveness of external precooling and vibration induced by Buzzy on pain and anxiety during inferior alveolar nerve block injection in children. Int J Dent.

[21] Simoncini E, Stiacini G, Morelli E, Trentini E, Peroni DG, Di Cicco M (2023). The effectiveness of the Buzzy device in reducing pain in children undergoing venipuncture: a single-center experience. Pediatr Emerg Care.

[22] Su HC, Hsieh CW, Lai NM, Chou PY, Lin PH, Chen KH (2021). Using vibrating and cold device for pain relief in children: a systematic review and meta-analysis of randomized controlled trials. J Pediatr Nurs.

[23] Baxter AL, Thrasher A, Etnoyer-Slaski JL, Cohen LL (2023). Multimodal mechanical stimulation reduces acute and chronic low back pain: pilot data from a HEAL phase 1 study. Front Pain Res (Lausanne).

[24] Nagpal D, Amlani DV, Rathi P, Hotwani KPS, Singh P, Gagandeep L (2023). Effect of audio distraction with thermomechanical stimulation on pain perception for inferior alveolar nerve block in children: a randomized clinical trial. J Dent Anesth Pain Med.

[25] Santos LM, Souza ER, Rocha PK, Maia EBS, Silva KEOP, Borges RS (2024). Effects of instructional therapeutic play in the behavior of children during the first attempt at intravenous catheterization. Rev Gaucha Enferm.

[26] Silva ACSS, Souza SL, Almeida LMM, Góes FGB, Knupp VMAO, Bonifácio MCS (2021). Clinical-epidemiological characterization of children and adolescents with congenital heart disease. Rev Pesqui (Univ Fed Estado Rio J Online).

[27] Shetty A, Naik SS, Patil RB, Valke PS, Mali S, Patil D (2023). Effectiveness of an extraoral cold and vibrating device in reducing pain perception during deposition of local anesthesia in pediatric patients aged 3-12 years: a split-mouth crossover study. J Dent Anesth Pain Med.

[28] Uzsen H, Buyuk ET, Odabasoglu E, Koyun M (2024). The effects of vibration and pressure interventions on children’s pain, fear and anxiety: a randomized controlled trial. J Pediatr Nurs.

[29] Guillari A, Giordano V, Catone M, Gallucci M, Rea T (2024). Non-pharmacological interventions to reduce procedural needle pain in children (6-12 years): a systematic review. J Pediatr Nurs.

[30] Palomares-González L, Hernández-Caravaca I, Gómez-García CI, Sánchez-Solís de Querol M (2023). Parental presence during invasive pediatric procedures: what does it depend on?. Rev. Latino-Am. Enfermagem.

