# Supplementary material for: Using Buzzy ® in venipunctures: perceptions of children and adolescents with congenital cardiopathies
Source: Rev Lat Am Enfermagem. 2026 Aug 10;34:e4849. doi: 10.1590/1518-8345.7822.4849 (PMC13459725; doi:10.1590/1518-8345.7822.4849)
Supplement: Translation

# Translation: **Uso do *Buzzy*** ^*®*^ em punção venosa: percepções de crianças e adolescentes com cardiopatias congênitas^*^

**DOI:** 10.1590/1518-8345.7822.4850

**Authors:** Lívia Grazielle Benevides dos Santos, Michelle Darezzo Rodrigues Nunes, Lucila Castanheira Nascimento, Liliane Faria da Silva, Fernanda Machado Silva-Rodrigues, Bárbara Bertolossi Marta de Araújo

**Affiliations:** 1 Hospital Federal de Bonsucesso, Rio de Janeiro, RJ, Brasil.; 2 Universidade do Estado do Rio de Janeiro, Faculdade de Enfermagem, Departamento Materno-Infantil, Rio de Janeiro, RJ, Brasil.; 3 Scholarship holder of the Programa de Incentivo à Produção Científica, Técnica e Artística da Universidade do Estado do Rio de Janeiro, Rio de Janeiro, RJ, Brasil.; 4 Universidade de São Paulo, Escola de Enfermagem de Ribeirão Preto, PAHO/WHO Collaborating Centre for Nursing Research Development, Ribeirão Preto, SP, Brasil.; 5 Universidade Federal Fluminense, Escola de Enfermagem Aurora de Afonso Costa, Departamento Materno-Infantil, Niterói, RJ, Brasil.; 6 Universidade de São Paulo, Escola de Enfermagem, São Paulo, SP, Brasil.

**Keywords:** Criança, Adolescente, Cardiopatias Congênitas, Dor, Manejo da Dor, Enfermagem Pediátrica.

## Abstract

**Objetivo:**

compreender a experiência de crianças e adolescentes com cardiopatias congênitas em relação ao uso da vibração associada à crioterapia (*Buzzy* ^*®*^ ) durante a punção venosa.

**Método:**

estudo qualitativo, descritivo e exploratório, com crianças e adolescentes hospitalizados com cardiopatias congênitas. A coleta de dados ocorreu por meio de entrevistas semiestruturadas audiogravadas, após uso do *Buzzy* ^*®*^ durante punção venosa. Os dados foram analisados mediante análise de conteúdo indutiva de Elo & Kyngas.

**Resultados:**

participaram 15 crianças e adolescentes, com média de idade de 11,1±3,6 anos. Foram construídas quatro categorias, que evidenciaram a experiência de crianças e adolescentes cardiopatas com o uso do *Buzzy* ^*®*^: Percebendo ou não a redução da dor; Vivenciando sensações físicas de relaxamento e anestesia; Distraindo-se com o *Buzzy* ^*®*^ ; e Reduzindo a ansiedade. Identificou-se que os participantes experienciaram redução da dor, de ansiedade, anestesia, relaxamento e distração com uso do dispositivo, sendo que apenas dois não perceberam diferença.

**Conclusão:**

o *Buzzy* ^*®*^ pode ser uma estratégia não farmacológica eficaz de gerenciamento da dor e ansiedade durante a punção venosa na clientela pediátrica, pois demonstrou benefícios nos aspectos físicos e emocionais, podendo ser utilizado tanto na prática clínica como em futuras pesquisas.

## Destaques

**(1)** As percepções sobre o uso do *Buzzy* ^*®*^ sugerem redução da dor em punção venosa. **(2)** Experiências dos participantes, adicionalmente, demonstram diminuição da ansiedade. **(3)** Importância da utilização de estratégias não farmacológicas em procedimentos.

## Introdução

As doenças cardíacas congênitas constituem uma das condições crônicas mais prevalentes na clientela infantojuvenil e, assim como em várias condições crônicas da infância, demandam frequentes idas ao hospital, com possibilidade de internações prolongadas e desgaste físico e emocional devido a exames físicos rotineiros preestabelecidos no plano terapêutico. Estar no ambiente hospitalar é estressante, pois a criança passa por tratamentos, alterações na rotina e múltiplos procedimentos dolorosos^1^. 

A dor, por si só, é um evento traumático para a criança, uma vez que causa intenso desconforto físico e repercussões nos aspectos emocionais, psicológicos e comportamentais. Diante da dor, elas frequentemente têm pensamentos fantasiosos, característicos da fase de desenvolvimento em que se encontram, nos quais dor e fatalidade podem ser consideradas sinônimos^2^. 

No conjunto de procedimentos invasivos executados com o uso de agulhas, a punção venosa está entre as principais causas de dor e angústia em pacientes pediátricos. A experiência nociceptiva aguda pode gerar repercussões psicológicas, emocionais e comportamentais indesejadas na percepção da dor^3^. 

Tal sensação está diretamente associada a consequências para crianças com malformações cardíacas, incluindo cianose grave com hipóxia tissular, débito cardíaco reduzido e alterações nutricionais. Esses efeitos são decorrentes da redução do fluxo sanguíneo pulmonar e do aumento dos hormônios estressores^4^.

Na maioria das vezes, a primeira alternativa para reduzir a dor e suas consequências em procedimentos dolorosos rotineiros realizados em crianças cardiopatas é o uso de fármacos como opioides e analgésicos^5^. No entanto, estudos demonstram que o metabolismo dos medicamentos é diretamente influenciado pelo estado cardíaco das crianças com cardiopatias, de modo que alterações de perfusão podem comprometer a metabolização do fármaco e aumentar o risco de intoxicação, bem como causar efeitos colaterais hemodinâmicos e respiratórios capazes de agravar ainda mais as condições de saúde^5^. 

Portanto, uma das alternativas na assistência a crianças e adolescentes com dor é o uso de métodos não farmacológicos, um deles o *Buzzy* ^*®*^ , um dispositivo tecnológico portátil de plástico em formato de abelha, joaninha ou borboleta, que combina o frio das asas congeláveis (crioterapia) com o estímulo vibratório do corpo. Tal dispositivo tem demostrado capacidade de reduzir o medo, a angústia e a ansiedade nas crianças, além de tranquilizar os pais e beneficiar os profissionais de saúde^6^. O efeito do *Buzzy* ^*®*^ baseia-se na “Teoria do controle de portão” descoberta por Melzack & Wall em 1965, a partir da qual é possível controlar as informações sinápticas da dor por intermédio da ativação de fibras nociceptivas. Esse mecanismo bloqueia a transmissão da sensação dolorosa das diferentes fibras receptivas à dor (fibras A-delta e C) por meio do frio e das vibrações emitidas pelo dispositivo^7^.

Com base na Teoria de Gerenciamento de Sintoma (TGS)^8^, referencial teórico que guiou o estudo, considera-se necessário manter o gerenciamento e controle da dor mesmo quando ela não é um sintoma, mas sim consequência de um procedimento invasivo. Dessa forma, a teoria pode auxiliar na identificação da experiência da dor e nas estratégias para minimizá-la.

Contudo, apesar de diversos ensaios clínicos randomizados mostrarem a eficácia do *Buzzy* ^*®(*^ ^6^, a literatura ainda carece de estudos qualitativos que investiguem a percepção da criança quanto ao uso deste dispositivo durante procedimentos agulhados^9^. Assim, entendendo que a voz de crianças e adolescentes é essencial para a proposição de qualquer tipo de intervenção com eles realizada, e reconhecendo-os como agentes morais, sujeitos de direitos e participantes ativos no processo de cuidado em saúde^10^, este estudo objetivou compreender a experiência de crianças e adolescentes com cardiopatias congênitas sobre o uso do *Buzzy* ^*®*^ durante a punção venosa. 

## Método

### Tipo de estudo

Estudo qualitativo, descritivo e exploratório. A pesquisa foi elaborada e apresentada de acordo com o *Consolidated Criteria for Reporting Qualitative Research (COREQ)* *.*

### Cenário e período de coleta de dados

Este estudo foi conduzido na enfermaria de cardiopediatria de um hospital federal, referência no tratamento de doenças cardíacas complexas, no Rio de Janeiro, Brasil. 

A coleta de dados transcorreu entre setembro de 2023 e março de 2024, até a saturação dos dados, ou seja, quando informações novas ou relevantes deixaram de surgir para o desenvolvimento das categorias e, portanto, os dados coletados foram considerados suficientes para a compreensão do fenômeno investigado^11^_._

### Participantes e critérios de seleção

Participaram do estudo 15 pacientes pediátricos que atendiam aos seguintes critérios de inclusão: crianças e adolescentes com cardiopatias congênitas na faixa etária entre seis e 18 anos incompletos, de ambos os sexos, hospitalizados no local de coleta de dados, submetidos a procedimento de punção venosa e que não apresentavam qualquer comprometimento cognitivo.

Punção venosa deve ser entendida como um procedimento no qual uma agulha é utilizada para penetrar na pele e nos vasos sanguíneos, a fim de viabilizar a coleta de amostras de sangue e tratamentos endovenosos através de acessos venosos periféricos^12^.

Foram excluídos do estudo crianças e adolescentes em cuidados paliativos, em situações críticas de internação ou que tivessem recebido algum tipo de sedativo ou analgésico para realização do procedimento, dada a possibilidade de vieses nos resultados. Também foi excluída a participação de crianças e adolescentes ainda sem definição diagnóstica e em precaução de contato, gotículas ou aerossóis, devido ao risco de contaminação cruzada com o uso do *Buzzy* ^*®*^ .

Crianças e adolescentes que atenderam aos critérios acima foram convidados a participar pessoalmente, e não houve qualquer recusa ou impossibilidade de participação.

### Procedimentos para a coleta de dados

Os dados de caracterização da criança e responsável (data de nascimento, sexo, diagnóstico, tratamento realizado e motivo da internação, por exemplo) foram coletados diretamente durante a entrevista ou por meio do prontuário. 

Após concordância em participar e assinatura do Termo de Consentimento Livre e Esclarecido, pelo responsável, e do Termo de Assentimento, pela criança ou adolescente, tiveram início os procedimentos para uso do *Buzzy* ^*®*^ XL (*Extra Large*), no formato de abelha, durante a punção venosa. Inicialmente, a enfermeira do setor e responsável pelo procedimento inspecionou os dois membros superiores da criança ou adolescente para escolha da veia e respectivo sítio de punção. 

Em seguida, cerca de um minuto antes da realização da punção, a pesquisadora acoplou as asinhas de silicone da abelha, armazenadas no refrigerador da enfermaria de cardiopediatria, ao corpo vibratório do *Buzzy* ^*®*^ . O dispositivo foi, então, posicionado sobre o ponto de entrada da agulha e fixado com o uso de uma faixa de silicone que vem junto com o dispositivo, para que o profissional tivesse as mãos livres para o procedimento. Uma vez posicionado e fixado, o aparelho foi ligado por 30 a 60 segundos. No momento da punção, o dispositivo foi deslocado cerca de cinco centímetros acima do local da picada e permaneceu ligado por mais, pelo menos, 60 segundos após término do procedimento. 

Para assegurar o rigor no desenvolvimento da pesquisa, as enfermeiras do setor de cardiopediatria que realizaram a punção venosa foram previamente instruídas sobre o dispositivo e sua função e receberam treinamento sobre a forma de aplicação e o passo a passo a ser seguido durante a coleta dos dados. Aspectos como tempo de aplicação sobre o ponto de entrada da agulha, distância de deslocamento do dispositivo em relação ao ponto de entrada da agulha e tempo de permanência do componente frio fora do refrigerador foram padronizados entre as profissionais, para evitar implicações nos resultados. A pesquisadora principal (LGBS) permaneceu presente durante a punção venosa em todas as coletas, auxiliando na aplicação do dispositivo.

Nos casos em que houve necessidade de uma segunda tentativa para obtenção do acesso periférico (P1, P9 e P14), o *Buzzy* ^*®*^ foi mantido sobre o local da primeira tentativa, até a identificação do novo sítio de punção pela enfermeira. Em seguida, foram seguidos os mesmos procedimentos para uso do *Buzzy* ^*®*^ XL no novo local.

Em alguns casos, após o procedimento, foi preciso aguardar alguns minutos para que as crianças ou adolescentes tivessem as sensações físicas da punção venosa atenuadas. Decorrido este período, iniciou-se a entrevista, e a criança ou adolescente foram convidados a responder algumas perguntas, dentre elas: “Como foi para você usar o *Buzzy* ^*®*^ no momento do procedimento?”, “Se 0 representa nenhuma dor e 10 o máximo de dor, qual o nível de dor com o uso da abelhinha, de 0 a 10?”, “Você já havia sido puncionado antes? Qual foi o nível de dor de 0 a 10 nessa experiência anterior?”. À medida que essas questões foram sendo respondidas, outras eram formuladas. Frases como *“Como assim?”* e *“Fale-me mais sobre isto”* buscaram aprofundar as respostas encontradas. Todas as crianças e adolescentes foram previamente instruídos acerca da escala numérica da dor. 

Antes e após cada uso, realizou-se a desinfecção do *Buzzy* ^*®*^ por meio da fricção com gaze umedecida com solução alcoólica a 70%. As entrevistas foram audiogravadas com aparelho celular e conduzidas pausadamente, de modo a observar o surgimento de algum desconforto ou emoção negativa por parte dos participantes, o que não foi identificado. Posteriormente, foram transcritas integralmente para leitura e análise. 

### Análise dos dados

Os dados qualitativos obtidos por meio das entrevistas semiestruturadas foram transcritos, e o conteúdo analisado de acordo com as etapas da análise de conteúdo indutiva, conforme os seguintes passos: preparação, organização e relato dos resultados^13^. 

A fase de preparação iniciou-se pela transcrição das respostas à entrevista semiestruturada. Nessa etapa, não há regras sistemáticas para análise de dados, porém as palavras do texto são agrupadas em categorias de conteúdo menores. Para isso, foram realizadas leituras minuciosas a fim de identificar o sentido contido nas respostas dos participantes e destrinchá-los. Na fase de organização, os dados foram codificados de forma aberta, agrupados e sintetizados com base em suas semelhanças e diferenças. E, por fim, as categorias foram construídas livremente. Fez-se o relato dos resultados mediante nomeação das categorias e subcategorias, de acordo com as características do conteúdo analisado^13^.

Com relação aos dados de caracterização da amostra, as variáveis dependentes nominais (sexo, diagnóstico, cor da pele, parentesco, escolaridade, estado civil, motivo da internação e procedência) foram analisadas e descritas em distribuição de frequência absolutas (n) e relativas (%), e as variáveis dependentes contínuas (idade e renda) relatadas por meio da análise das medidas de tendência central (média) e dispersão (desvio-padrão)^14^.

### Aspectos éticos

O projeto foi submetido e aprovado pelo Comitê de Ética e Pesquisa com Seres Humanos (CEP) das instituições proponente e coparticipante, mediante processo nº 6.132.990 e CAAE nº 68806623.9.0000.5282. Todas as etapas contemplaram as diretrizes presentes na Resolução do Conselho Nacional de Saúde 466/2012^15^.

Nos procedimentos para a coleta dos dados, após a identificação dos participantes que atenderam aos critérios de seleção, foram apresentados aos responsáveis o tema e os objetivos da pesquisa. Após concordância em participar, houve assinatura do Termo de Consentimento Livre e Esclarecido, pelo responsável, e do Termo de Assentimento, pela criança ou adolescente. As informações obtidas por meio deste estudo foram tratadas de forma confidencial, sendo assegurado o sigilo por meio do uso da codificação, em que “P” se refere à inicial da palavra “Participante”, e os respectivos números, de 1 a 15, correspondem à ordem de realização das entrevistas, seguidos da idade. 

Os participantes receberam orientações sobre seus direitos, objetivos da pesquisa e risco mínimo de desconforto emocional durante a entrevista, dado o momento de fragilidade por eles vivenciado durante a hospitalização. Também foram orientados acerca da possibilidade de interromperem suas participações a qualquer momento, sem qualquer prejuízo.

Declara-se ausência de conflito de interesses no uso do *Buzzy* ^*®*^ System nesta pesquisa.

## Resultados

Participaram do estudo 15 crianças e adolescentes com cardiopatias congênitas. A média de idade dos participantes foi de 11,1±3,6 anos, sendo que sete (n=7) eram crianças entre seis e 11 anos (47%) e oito (n=8) adolescentes entre 12 e 17 anos (53%). A maioria era do sexo masculino (n=8, 53%) e estava internada para tratamento cirúrgico (n=13, 86%). No que se refere ao diagnóstico, predominaram as cardiopatias acianóticas (n=6, 40%). 

Todos os participantes foram puncionados com cateter flexível de diâmetro 22 ou 24, de acordo com a avaliação da enfermeira responsável pelo procedimento. As punções foram realizadas nos membros superiores, na fossa antecubital (n= 4), no dorso da mão (n=7) ou no antebraço (n=4). Em apenas três punções (n=3) foram necessárias duas tentativas para obtenção do acesso.

Dos 15 participantes, 13 referiram dor menor com o uso do *Buzzy* ^*®*^ e dois avaliaram a dor da punção como sendo da mesma intensidade com o uso do dispositivo. 

Após análise das entrevistas, foram elaboradas quatro categorias e uma subcategoria, apresentadas a seguir. 

### Percebendo ou não a redução da dor

Quando convidados a responder perguntas sobre a experiência de dor com o uso do *Buzzy* ^*®*^ , a maioria dos participantes referiu que, graças ao dispositivo, não foi possível sentir por completo a entrada da agulha no momento da punção, assim considerando a dor amenizada. Segundo eles, isso contribuiu para que a experiência da punção venosa apresentasse menor desconforto, com consequências positivas para a punção venosa de maneira geral, por evitar o choro e a movimentação abrupta do membro na hora do procedimento. Além disso, demonstraram-se encantados com a diminuição da sensibilidade e consequente redução da sensação de dor.

*A abelhinha não doeu. Eu acho que eu não vou chorar mais [...] eu gostei muito dela, vou dar um beijinho nela! (P1, 9 anos).*

*[...] Na minha experiência né, eu senti entrando a agulha, mas eu não senti a picada por causa da vibração [...] ajuda muito que você também não sente a agulha e aí a pessoa acaba ficando encantada igual eu (P4, 13 anos).*

*Porque deixou o braço daquele jeito sem vida, aí não ardeu, o gelado ajuda junto com a vibração, dá pra sentir, mas é pouco. A dor é praticamente nada (P13, 13 anos).*

*Foi diferente, eu nem puxei a mão na hora que ela (enfermeira) pegou, porque eu nem senti a agulha direito, foi rápido e tranquilo [...] foi sem eu sentir muito desconforto. Eu senti o aparelhinho né, foi bom porque parece que a dor deu uma amenizada (P15, 12 anos).*

Para os participantes, o *Buzzy* ^*®*^ foi capaz de reduzir significativamente a sensação de dor da picada na hora da punção venosa em comparação com os procedimentos agulhados realizados anteriormente à pesquisa, sem o uso do aparelho.

*Pesquisadora: De 0 a 10, quanto que você diria que foi sua dor nessas outras experiências? Adolescente: Eu acho que foi oito ou nove. Pesquisadora: E aí agora, depois de usar a abelhinha, de 0 a 10 quanto foi sua dor? Adolescente: Três (P4, 13 anos).*

*Pesquisadora: E de 0 a 10, quanto foi sua dor nessas outras injeções que você já tomou antes? 0 significa nenhuma dor e 10 é muita dor. Adolescente: Doeu muito. Uns oito. Pesquisadora: E com o uso da abelhinha, de 0 a 10, você achou que a injeção doeu quanto? Adolescente: Só dois (P6, 15 anos).*

*Pesquisadora: Se você fosse dar uma nota em que 0 não arde nada, 1 arde um pouco, 2 arde um pouquinho mais, 3 já arde mais... até chegar no 10, que arde muito, qual nota você daria? Criança: Dez. Pesquisadora: Entendi, e hoje com a abelhinha? Qual nota você dá? Criança: Um (1) (P11, 8 anos).*

Dois (n=2) participantes mencionaram experiências equivalentes com relação à intensidade da dor com e sem a abelhinha, sendo estes os mesmos que precisaram de duas punções para sucesso do procedimento. Segundo eles, a dor com a utilização do dispositivo durante a punção venosa foi bem semelhante à sentida em experiências passadas.

*Pesquisadora: Então, de 0 a 10, quanto foi a dor usando a abelhinha? Adolescente: Bom, vou dar a mesma nota de tirar sangue normal, uns 5 ou 6 (P9, 13 anos).*

*Pesquisadora: E que nota você daria, de 0 a 10, pra dor que você sentiu quando a agulha entrou e você estava com a abelhinha? Criança: Dou 7. Pesquisadora: E sem a abelhinha nessas outras vezes? Criança: 7 também. Foi bem parecido, doeu do mesmo jeito (P14, 9 anos).*

### **Destacando possibilidades de melhorias no uso do *Buzzy*** ^®^

As percepções de alguns participantes sobre o uso do dispositivo auxiliaram no reconhecimento de algumas limitações e sugestões de melhorias para utilização desta ferramenta, a fim de potencializar a redução da dor. Dois participantes (P5 e P9) destacaram que o componente frio poderia ser mais eficaz se o tempo de congelamento fosse maior, e outro (n=1) que a vibração tinha baixa intensidade e, portanto, era pouco percebida. Destaca-se que esses dois participantes foram os mesmos que necessitaram de mais de uma punção para o sucesso do procedimento, o que demandou maior tempo para a realização do procedimento.

*Se ela estivesse um pouco mais gelada, eu acho que conseguiria anestesiar melhor. Eu não senti a vibração. [...] só senti as asas dela (P9, 13 anos).*

*[...] Se a abelhinha estivesse um pouco mais congelada...porque ela estava meio líquida, né? (P5, 11 anos).*

### Vivenciando sensações físicas de relaxamento e anestesia

Através das falas dos participantes, observou-se que o *Buzzy* ^*®*^ é capaz de proporcionar sensações de relaxamento e anestesia. Para eles, o gelo e a vibração, em contato com a pele, desencadeiam sensações de relaxamento muscular, dormência, formigamento e anestesia no membro puncionado. Por isso, sentem que o local puncionado é massageado, paralisado ou ainda adormecido e sem vida. Como consequência, ficam com o local da punção menos sensível durante o procedimento, o que os ajuda a enfrentar o tão temido momento da punção.

*É bom. [...] Porque relaxa. [...] A gente sente um relaxamento que fica bom (P7, 9 anos).*

*Ficava vibrando, né? Aí ficava mexendo, aí relaxava um pouco (...) O braço, sabe? Aí eu senti meio que o braço um pouco anestesiado, mas só no braço. A anestesia é meio que você sentir o braço um pouco como se fosse dormente e não sentir dor na região. Porque o gelo anestesia quando coloca ele sobre algum lugar. Como anestesiou aqui né (aponta o local de posicionamento do Buzzy* ^*®*^ ) (P9, 13 anos).

*Ela fez não doer, senti formigamento e mais nada (P10, 6 anos) (...) não doeu nada, por causa daquela sensação que formiga e treme, faz não sentir a agulha normal (P10, 6 anos).*

*Quando colocou gelo ficou dormente pra colocar a agulha e nem pareceu que tinha agulha. É porque o gelo deixou mais dormente a mão, e não deu pra sentir muito a agulha, só senti uma picada bem fininha (P12, 13 anos).*

*Porque ela mexe daquele jeito diferente, o braço formiga na hora e fica paralisado um pouco. A sensação que dá que ele fica menos sensível (P14, 9 anos).*

### **Distraindo-se com o *Buzzy*** ^®^

As crianças e adolescentes também fizeram comentários a respeito da aparência do *Buzzy* ^*®*^ , utilizando palavras como bonito, interessante, parecido com um brinquedo, alegre e feliz. Esses adjetivos foram relacionados à distração no momento da realização do procedimento com agulha, que costuma ser tão estressante. Nos relatos, identificou-se ainda o desejo de interagir com o próprio dispositivo.

Eles avaliaram a aparência do instrumento como adjuvante para proporcionar calma, distração e felicidade, e relacionaram a forma de abelha à familiaridade com os desenhos presentes em seus cotidianos. Todas essas características, para esse público, conferem simpatia ao aparelho e ajudam a entretê-los e fazer com que se desconcentrem da punção venosa.

*Ela é bonitinha. Eu achei muito interessante porque a aparência, com certeza, vai acalmar a criança, ela vai achar bonitinho, vai fazer uma distração. Vai fazer com que a criança não pense na agulha, mas sim na abelhinha. (P2, 14 anos).*

*Porque é bonitinho, o rostinho dela, é simpática [...] ela sorri, fica alegre (P14, 9 anos).*

*Ela é igual a um brinquedo (...) Por causa do barulhinho. Por causa da asa também! É bom também porque tem a forma dela. Tem esses desenhos. O rostinho sorrindo. [...] É igual ao desenho que a gente faz na escola, mas a gente não consegue fazer a carinha assim. (...) Feliz e contente. Deixa ela mais bonita e dá vontade de pegar nela. [...] distrai a cabeça. [...] ajuda a não pensar na agulha (P10, 6 anos).*

Apesar disso, a mesma criança da fala anterior trouxe, como contraponto, a possibilidade de que a aparência do *Buzzy* ^*®*^ pudesse provocar sentimentos contraditórios, já que abelhas podem dar picadas dolorosas.

*Criança: Na hora que tremeu, eu achei que a abelhinha estava com a agulha, mas depois eu vi que não e que parou de doer. Pesquisadora: E por que você achou que ela tinha agulha? Criança: Porque abelha tem aquele negócio que pica (P10, 6 anos).*

### Reduzindo a ansiedade

Através dos relatos, observou-se que o *Buzzy* ^*®*^ reduz a ansiedade associada à punção venosa, por dois motivos: crianças e adolescentes depositam expectativas quanto à redução da dor ao utilizarem o aparelho e os próprios recursos sensoriais do dispositivo (vibração e frio) favorecerem a analgesia. Assim, ao voltarem suas atenções para o dispositivo e perceberem a redução da sensação dolorosa, deixam de pensar na punção e ficam mais calmos e relaxados. Em suas experiências, o medo da agulha, o nervosismo e o susto atrelados ao procedimento foram minimizados com o uso do *Buzzy* ^*®*^ . 

Por resultar na redução da tensão em um momento de vulnerabilidade, elogiaram a aplicação do dispositivo como uma intervenção muito boa, encantadora e com potencial de acalmá-los.

*[...] O fato de a gente imaginar que não vai doer tanto também acalma bastante, tira aquele nervosismo que normalmente atrapalha na hora (P2, 14 anos).*

*Ai, eu fiquei encantada! Eu acho que pra quem tem muito medo de agulha, assim como eu, pra quem tem aflição ou tem aflição de sangue, na hora também é muito bom (P4, 13 anos).*

*Quando você colocou a abelha, deixou vibrando, já ficou melhor porque já acalmou [...]ela acalma porque faz não sentir tanta dor assim. Eu sempre tive medo de agulha né, mas...não tinha jeito, e parecia que estava beliscando infinito até tirar a agulha. E, com a abelha, eu senti muito melhor (P12, 13 anos).*

*Porque doeu menos na hora, o susto foi menor. Na hora que entrou a agulha, como eu não senti tanto igual antes, eu não tive aquela reação de susto na hora [...] tinha que ter isso das outras vezes pra ajudar a diminuir esse susto (P15, 12 anos).*

## Discussão

As percepções e experiências subjetivas dos participantes indicam que o dispositivo *Buzzy* ^*®*^ reduz a dor e a ansiedade durante procedimentos agulhados em crianças e adolescentes com cardiopatias congênitas, por gerar sensações de anestesia e formigamento, promover o relaxamento da musculatura e favorecer a distração.

Embora seja comum a necessidade de punções venosas nas unidades de internação para realização de exames e do tratamento proposto, trata-se de um procedimento que gera dor e, por isso, causa medo, ansiedade, estresse físico e emocional^16^. Todos esses sentimentos e estressores vivenciados de forma contínua podem contribuir para danos no crescimento e desenvolvimento de crianças e adolescentes, uma vez que afetam o sistema nervoso central, a cognição e o aprimoramento motor e emocional. Ademais, podem comprometer a aderência de crianças e adolescentes a cuidados de saúde no futuro, o que torna esta temática de extrema importância^17^.

Apesar de muitos avanços na avaliação e tratamento da dor no público pediátrico, ainda há dificuldade em quantificá-la por seu caráter subjetivo, bem como tratá-la adequadamente. Como consequência, crianças e adolescentes cardiopatas vivenciam o sofrimento emocional e sensitivo, com riscos de passarem por alterações hemodinâmicas importantes, com variações de oxigenação tecidual por oscilações de pressão intravascular^18^. Logo, é preciso encontrar uma forma de amenizar a experiência dolorosa física e emocional, inclusive durante procedimentos com agulha, a fim de humanizar a hospitalização e minimizar os impactos negativos sobre a qualidade de vida^19^.

Recente revisão de literatura^18^ investigou as principais intervenções não farmacológicas para manejo da dor em crianças maiores de um ano submetidas a procedimentos com agulha e concluiu que os três principais métodos adotados para punção venosa periférica foram goma de mascar, distração e o *Buzzy* ^*®*^ .

Entende-se, com base na Teoria do Gerenciamento dos Sintomas (TGS), referencial teórico que guiou este estudo^7^, que as respostas a sintomas como a dor podem englobar dimensões fisiológicas, psicológicas e comportamentais e que a melhor maneira de compreender essa experiência é por meio do autorrelato do indivíduo que a vivencia. Logo, buscou-se uma abordagem mais precisa e aprofundada sobre esse fenômeno do ponto de vista qualitativo, sob a perspectiva de crianças e adolescentes com cardiopatias congênitas, no intuito de explorar os significados por eles atribuídos às avaliações do uso deste dispositivo e formas como respondem ao sintoma após a aplicação da intervenção proposta. 

Nos relatos, constatou-se que a intervenção resultou em diminuição da dor para quase todos os participantes, exceto para dois deles, que a perceberam inalterada. Isso pode ser explicado pela relação existente entre os mecanismos para o efeito de analgesia local gerados durante a punção e o compartilhamento de sinapses na medula espinhal, em que as fibras de condução do estímulo doloroso compartilham as vias com as fibras de condução térmica e mecânica. Essa interferência interneural na condução sináptica é capaz de gerar a sensação de relaxamento mencionada pelos participantes e, por conseguinte, o alívio da dor^20^.

A modulação da dor com o uso *Buzzy* ^*®*^ ocorre pela influência do dispositivo tecnológico no sistema inibitório descendente, de origem na substância cinzenta do mesencéfalo. Nesse caso, a informação dolorosa que partiria do encéfalo em direção ao corno dorsal da medula espinhal é diminuída em decorrência de alterações serotoninérgicas^21^. Como resultado, a dor física e o sofrimento emocional são amenizados com o uso do *Buzzy* ^*®*^ . 

Além dos benefícios para os pacientes, o procedimento torna-se menos traumático para o profissional que punciona, pois pode ser realizado de forma mais rápida e tranquila, com menores chances de choro, agitação e movimentos abruptos. Logo, o uso do *Buzzy* ^*®*^ pode tornar o procedimento mais seguro e aumentar a chance de sucesso do profissional na primeira tentativa de punção. Sensações atribuídas ao dispositivo, como “não chorar”, “se acalmar”, “estar menos nervosa” e mais “relaxada”, auxiliam nesse processo.

Adicionalmente, a redução da dor veio acompanhada, nos relatos, de sensações de “anestesia”, “relaxamento”, “dormência” ou “adormecimento”, “paralisia” e “formigamento”. A combinação da crioterapia com a vibração tem eficácia potencializada também pela liberação de opioides endógenos decorrentes da ativação de receptores de toque relacionados ao frio, o que causa uma sensação de entorpecimento local, com consequente “anestesia”, conforme relatos dos participantes^21^.

Estudo clínico elaborado pela desenvolvedora da tecnologia em questão comparou diferentes vibrações de frequências para determinar qual seria melhor aplicada ao aparelho. Verificou-se que a frequência entre 180-200 Hz aplicada à tecnologia Oscillice do *Buzzy* ^*®*^ é potente o bastante para ultrapassar os efeitos de terapias à base de estimulação elétrica na redução de dores pós-cirúrgicas, coluna e outras lesões^22^. Em concordância com esses resultados, outro estudo demonstrou que a vibração mecânica de alta frequência proporciona alívio significativo da dor musculoesquelética, mostrando-se mais eficaz que métodos tradicionais, inclusive farmacológicos, o que corrobora os relatos dos participantes deste estudo^23^.

Ainda sobre as sensações referidas pelas crianças e adolescentes, faz-se importante ressaltar que o resfriamento local obtido pelas asinhas de gelo proporciona analgesia e contribui para o entorpecimento mediante diminuição da taxa metabólica local e redução do fluxo de mediadores inflamatórios. A interrupção da transmissão dos sinais da dor por essa via aumenta sucessivamente o limiar absoluto da dor com o aumento de betaendorfinas e, em pouco tempo, os músculos tornam-se “relaxados” e “adormecidos”, facilitando o procedimento agulhado^24^. Além dos elementos previamente discutidos sobre o aparelho que geram analgesia, a distração é um dos mecanismos que conferem efetividade ao *Buzzy* ^*®*^ e vem sendo atestada em diversos ensaios clínicos randomizados^21^.

O traço lúdico do dispositivo remete ao cenário de brincadeira para a criança e está intimamente ligado ao desenvolvimento emocional e cognitivo. Segundo estudo, quando o lúdico é usado corretamente pelo profissional através do cuidado participativo, é capaz de ajudar a criança a estruturar os acontecimentos a sua volta, por reduzir a ansiedade, aumentar a aceitação do tratamento, melhorar a comunicação e auxiliar na ressignificação do momento de hospitalização^25^. 

Em artigos internacionais, identificou-se que, na vigência de quadros de ansiedade em procedimentos agulhados, crianças e adolescentes podem apresentar aumento considerável da frequência cardíaca^21^ ^,^ ^26^ ^-^ ^28^, além de hipoxemia, alterações vasovagais e hormonais^21^ ^,^ ^28^. Com o uso *Buzzy* ^*®*^ , percebeu-se redução dos parâmetros cardíacos para níveis próximos da normalidade^26^ ^-^ ^27^.

Embora crianças e adolescentes reconheçam o *Buzzy* ^*®*^ como um instrumento útil para reduzir a dor processual, os achados revelam aspectos do dispositivo que representam desafios e possibilidades de aprimoramento. Nesse sentido, considera-se importante desenvolver protocolos mais adaptativos, que levem em conta variáveis desenvolvimentais, sensoriais e contextuais, a fim de otimizar a experiência desses indivíduos^26^.

Neste estudo, dois participantes não referiram alívio da sensação dolorosa. Apesar de a maioria dos estudos demonstrarem a eficácia do dispositivo, outras pesquisas corroboram a necessidade de estratégias individualizadas^29^. Sublinha-se que ambos os participantes são os mesmos que não obtiveram sucesso no acesso periférico na primeira tentativa. Além disso, um deles mencionou a oportunidade de melhoria com relação ao tempo de congelamento das asinhas. De acordo com estudo^25^, tentativas repetidas de cateterização periférica podem aumentar a sensibilidade da criança e do adolescente a esse tipo de procedimento e potencializar a dor, pois procedimentos com agulhas são a primeira causa de medo nesses pacientes^25^. 

Outro participante que precisou de uma segunda punção referiu alívio da sensação dolorosa durante o procedimento. Assim, acredita-se que diversos fatores possam influenciar a sensação dolorosa, tais como estágio de desenvolvimento da criança^7^ ^,^ ^28^, maior dificuldade de modulação da dor em crianças menores de sete anos, variações na aplicação da técnica, tempo de exposição à crioterapia, preferências individuais quanto a cores e formas que podem não ressoar com a apresentação do dispositivo^24^ ^,^ ^26^ ^)^ e presença parental^30^. 

Contraditoriamente à visão lúdica do dispositivo, houve uma associação do formato de abelha do *Buzzy* ^*®*^ com risco iminente de uma sensação dolorosa, relacionada à picada do inseto. Crianças mais novas ainda incorporam elementos fantasiosos em suas realidades^20^, o que pode tornar a abelhinha assustadora para algumas. Nesse caso, optar pela variação joaninha seria uma opção.

Ademais, dois participantes consideraram insuficiente o tempo de congelamento das asas da abelhinha para proporcionar a analgesia adequada, já que, durante a punção, já estavam em estado líquido. Sabe-se que o tempo de descongelamento do componente frio, a partir do momento em que é retirado do freezer, é de 10 minutos^23^. Isso significa que, para procedimentos mais longos ou com maior demora para operacionalização, seria importante retirar a asinha do refrigerador o mais próximo possível do início ou substituí-la por outra.

É possível destacar, como limitações do estudo, a necessidade de formato mais adequado do *Buzzy* ^*®*^ de acordo com a faixa etária dos usuários, bem como a substituição das asas de silicone geladas quando ficarem fora do refrigerador por mais de 10 minutos. 

Dar voz à criança e ao adolescente é uma importante estratégia de empoderamento e inclusão no processo de cuidado, pois facilita a aproximação dessa clientela da equipe de saúde e do saber científico. Paralelamente, estudos reforçam que o relato da própria criança e adolescente sobre sua situação de saúde ou sobre suas vivências é a melhor maneira de compreender suas visões, tornando cada fala extremamente valiosa para compreensão do todo^8^ ^,^ ^10^ ^,^ ^25^.

Ressalta-se, como potencialidade deste estudo, o incentivo à utilização de intervenções não farmacológicas pela equipe de saúde, algo que ainda é escasso na prática clínica. Isso pode ser percebido, por exemplo, no estudo que avaliou 1.251 prontuários de crianças hospitalizadas e identificou, em apenas seis deles, anotações de enfermagem sobre o uso de medidas não farmacológicas para gerenciamento de dor, tais como calor local, distração e compressa fria^2^. Entende-se que a TGS, por ser composta por três componentes [a experiência do sintoma - neste estudo a dor; as estratégias para o gerenciamento do sintoma (*Buzzy* ^*®*^ ); e os resultados - dor com uso do *Buzzy* ^*®*^ ], tem potencial para, além de guiar intervenções de enfermagem com vistas ao adequado gerenciamento de sintomas na prática, influenciar as práticas institucionais e criar ambientes de cuidados propícios para potencializar a qualidade de vida dessa população^8^.

Novas pesquisas sobre o uso do *Buzzy* ^*®*^ em crianças e adolescentes submetidos a procedimentos dolorosos e com uso de agulhas devem ser realizadas para aprofundar esses achados e investigar a experiência desse público pediátrico em uma variedade maior de procedimentos, situações de tratamento (intra e extra-hospitalar) e diagnósticos e sob diferentes delineamentos (como estudos clínicos randomizados ou abordagens mistas). Dessa forma, será possível aprofundar a compreensão dos efeitos do *Buzzy* ^*®*^ . 

## Conclusão

As quatro categorias encontradas (“Percebendo ou não a redução da dor”; “Vivenciando sensações físicas de relaxamento e anestesia”; “Distraindo-se com o *Buzzy* ^*®*^ ”; e “Reduzindo a ansiedade”) evidenciam a experiência de crianças e adolescentes cardiopatas com o uso do *Buzzy* ^*®*^ durante a punção venosa. Na avaliação dos participantes, trata-se de uma intervenção não farmacológica eficaz, interessante e encantadora, que atua tanto em suas dimensões físicas quanto emocionais, pois experimentam menos dor, ansiedade e nervosismo durante o procedimento, com maior relaxamento e distração. 

Acredita-se que os resultados deste estudo possam contribuir para a pesquisa, dada a redução por eles proporcionada na lacuna nacional e internacional evidenciada durante busca na literatura. Adicionalmente, auxiliam na sensibilização de outros profissionais acerca da importância da utilização de estratégias de redução da dor durante punção venosa, de modo a qualificar a assistência de enfermagem a crianças e adolescentes e tornar o atendimento humanizado e menos traumatizante.

## Data Availability

Os conjuntos de dados relacionados a este artigo estarão disponíveis mediante solicitação ao autor correspondente.

# Translation: **Uso de *Buzzy*** ^*®*^ en venopunción: percepciones de niños y adolescentes con cardiopatías congénitas^*^

**DOI:** 10.1590/1518-8345.7822.4848

**Authors:** Lívia Grazielle Benevides dos Santos, Michelle Darezzo Rodrigues Nunes, Lucila Castanheira Nascimento, Liliane Faria da Silva, Fernanda Machado Silva-Rodrigues, Bárbara Bertolossi Marta de Araújo

**Affiliations:** 1 Hospital Federal de Bonsucesso, Rio de Janeiro, RJ, Brasil.; 2 Universidade do Estado do Rio de Janeiro, Faculdade de Enfermagem, Departamento Materno-Infantil, Rio de Janeiro, RJ, Brasil.; 3 Scholarship holder of the Programa de Incentivo à Produção Científica, Técnica e Artística da Universidade do Estado do Rio de Janeiro, Rio de Janeiro, RJ, Brasil.; 4 Universidade de São Paulo, Escola de Enfermagem de Ribeirão Preto, PAHO/WHO Collaborating Centre for Nursing Research Development, Ribeirão Preto, SP, Brasil.; 5 Universidade Federal Fluminense, Escola de Enfermagem Aurora de Afonso Costa, Departamento Materno-Infantil, Niterói, RJ, Brasil.; 6 Universidade de São Paulo, Escola de Enfermagem, São Paulo, SP, Brasil.

**Keywords:** Niño, Adolescente, Cardiopatías Congénitas, Dolor, Manejo del Dolor, Enfermería Pediátrica

## Abstract

**Objetivo:**

comprender la experiencia de niños y adolescentes con cardiopatías congénitas con respecto al uso de vibración combinada con crioterapia (*Buzzy* ^*®*^ ) durante la venopunción.

**Método:**

estudio cualitativo, descriptivo y exploratorio con niños y adolescentes hospitalizados con cardiopatías congénitas. La recopilación de datos se realizó mediante entrevistas semiestructuradas grabadas en audio tras el uso de *Buzzy* ^*®*^ durante la venopunción. Los datos se analizaron mediante el análisis de contenido inductivo de Elo y Kyngas.

**Resultados:**

participaron 15 niños y adolescentes, con una edad media de 11,1±3,6 años. Se construyeron cuatro categorías que destacaron la experiencia de niños y adolescentes con cardiopatías con *Buzzy* ^*®*^: Percepción o no de una reducción del dolor; Presencia de sensaciones físicas de relajación y anestesia; Distracción con *Buzzy* ^*®*^ ; y Reducción de la ansiedad. Se identificó que los participantes experimentaron una reducción del dolor, la ansiedad, la anestesia, además de la relajación y la distracción con el uso del dispositivo; advirtiéndose que solo dos no percibieron ninguna diferencia.

**Conclusión:**

el uso de Buzzy^®^ puede ser una estrategia no farmacológica eficaz para el manejo del dolor y la ansiedad durante la venopunción en pacientes pediátricos, ya que ha demostrado beneficios en aspectos físicos y emocionales, por lo que puede ser utilizado en la práctica clínica, así como en futuras investigaciones.

## Destacados

**(1)** Las percepciones sobre el uso de *Buzzy* ^*®*^ sugieren una reducción del dolor durante la venopunción. **(2)** Las experiencias de los participantes también demuestran una disminución de la ansiedad. **(3)** Se destaca la importancia de utilizar estrategias no farmacológicas en los procedimientos.

## Introducción

Las cardiopatías congénitas constituyen una de las afecciones crónicas más prevalentes en la población infantil y juvenil y, al igual que otras afecciones crónicas de la infancia, requieren frecuentes visitas al hospital, con la posibilidad de hospitalizaciones prolongadas y desgaste físico y emocional debido a los exámenes físicos rutinarios preestablecidos en el plan terapéutico. Estar en el entorno hospitalario es estresante, ya que el niño se somete a tratamientos, cambios en la rutina y múltiples procedimientos dolorosos^1^.

En sí mismo, el dolor es un evento traumático para el niño, ya que causa un intenso malestar físico y repercute en los aspectos emocionales, psicológicos y conductuales. Ante el dolor, suelen tener pensamientos fantásticos, característicos de la etapa de desarrollo en la que se encuentran, en la que dolor y fatalidad pueden considerarse sinónimos^2^.

En el conjunto de los procedimientos invasivos realizados con agujas, la venopunción se encuentra entre las principales causas de dolor y angustia en pacientes pediátricos. La experiencia nociceptiva aguda puede generar repercusiones psicológicas, emocionales y conductuales indeseables en la percepción del dolor^3^.

Esta sensación está directamente relacionada con consecuencias para los niños con malformaciones cardíacas, como cianosis grave con hipoxia tisular, disminución del gasto cardíaco y alteraciones nutricionales. Estos efectos son consecuencia de la reducción del flujo sanguíneo pulmonar y el aumento de las hormonas del estrés^4^.

En la mayoría de los casos, la primera alternativa para reducir el dolor y sus consecuencias en procedimientos dolorosos rutinarios realizados en niños con cardiopatías es el uso de fármacos como opioides y analgésicos^5^. Sin embargo, estudios demuestran que el metabolismo de los fármacos se ve directamente influenciado por el estado cardíaco de los niños con cardiopatías, por lo que las alteraciones de la perfusión pueden comprometer el metabolismo de los fármacos y aumentar el riesgo de intoxicación, además de causar efectos colaterales hemodinámicos y respiratorios que pueden agravar aún más las condiciones de salud^5^.

Por lo tanto, una de las alternativas para ayudar a niños y adolescentes con dolor es el uso de métodos no farmacológicos, como *Buzzy* ^*®*^ , un dispositivo tecnológico portátil de plástico con forma de abeja, mariquita o mariposa, que combina el frío de las alas congelables (crioterapia) con la estimulación vibratoria corporal. Este dispositivo ha demostrado su capacidad para reducir el miedo, la angustia y la ansiedad en niños, además de tranquilizar a los padres y beneficiar a los profesionales sanitarios^6^. El efecto de *Buzzy* ^*®*^ se basa en la “Teoría de la compuerta” descubierta por Melzack y Wall en 1965, según la cual es posible controlar la información sináptica del dolor mediante la activación de fibras nociceptivas. Este mecanismo bloquea la transmisión de la sensación dolorosa desde diferentes fibras receptoras del dolor (fibras A-delta y C) mediante el frío y las vibraciones emitidas por el dispositivo^7^.

Con base en la Teoría del Manejo de Síntomas (TMS)^8^, marco teórico que guió el estudio, se considera necesario mantener el manejo y control del dolor incluso cuando no es un síntoma, sino una consecuencia de un procedimiento invasivo. De esta manera, la teoría puede ayudar a identificar la experiencia del dolor y a desarrollar estrategias para minimizarlo.

Sin embargo, a pesar de varios ensayos clínicos aleatorizados que muestran la efectividad de *Buzzy* ^*®(*^ ^6^, la literatura aún carece de estudios cualitativos que investiguen la percepción del niño sobre el uso de este dispositivo durante los procedimientos con agujas^9^. Así, entendiendo que la voz de los niños y adolescentes es esencial para proponer cualquier tipo de intervención realizada con ellos, y reconociéndolos como agentes morales, sujetos de derechos y participantes activos en el proceso de atención a la salud^10^, este estudio tuvo como objetivo comprender la experiencia de niños y adolescentes con cardiopatías congénitas en relación al uso de *Buzzy* ^*®*^ durante la venopunción.

## Método

### Tipo de estudio

Estudio cualitativo, descriptivo y exploratorio. La investigación se diseñó y presentó de acuerdo con los *Consolidated Criteria for Reporting Qualitative Research* (COREQ).

### Escenario y periodo de recolección de datos

Este estudio se realizó en la sala de cardiología pediátrica de un hospital federal, un centro de referencia para el tratamiento de enfermedades cardíacas complejas, en Río de Janeiro, Brasil.

La recolección de datos se realizó entre septiembre de 2023 y marzo de 2024, hasta la saturación de datos, es decir, cuando ya no surgía información nueva o relevante para el desarrollo de las categorías y, por lo tanto, los datos recolectados se consideraron suficientes para la comprensión del fenómeno investigado^11^.

### Participantes y criterios de selección

Se incluyeron en el estudio 15 pacientes pediátricos que cumplieran los siguientes criterios de inclusión: niños y adolescentes con cardiopatía congénita, con edad entre seis y 18 años (no cumplidos), de ambos sexos, hospitalizados en el sitio de recolección de datos, sometidos a venopunción y que no presentaban deterioro cognitivo.

La venopunción debe entenderse como un procedimiento en el que se utiliza una aguja para penetrar la piel y los vasos sanguíneos con el fin de posibilitar la recolección de muestras de sangre y tratamientos intravenosos a través del acceso venoso periférico^12^.

Se excluyó del estudio a niños y adolescentes en cuidados paliativos, en situación de hospitalización crítica o que hubieran recibido algún tipo de sedante o analgésico para el procedimiento, dada la posibilidad de sesgo en los resultados. También se excluyó a niños y adolescentes sin diagnóstico definido y con precauciones de contacto, gotículas o aerosoles debido al riesgo de contaminación cruzada con el uso de *Buzzy* ^*®*^ .

Los niños y adolescentes que cumplieron con los criterios antes mencionados fueron invitados a participar en persona y no hubo rechazos ni imposibilidades que impidieran la participación.

### Procedimientos para la recopilación de datos

Los datos que caracterizan al niño y al cuidador (fecha de nacimiento, sexo, diagnóstico, tratamiento recibido y motivo de hospitalización, por ejemplo) se recogieron directamente durante la entrevista o a través de la historia clínica.

Tras haber aceptado la participación, y luego de la firma del Formulario de Consentimiento Informado por el responsable, así como del Formulario de Asentimiento por parte del niño o adolescente, se iniciaron los procedimientos para el uso de *Buzzy* ^*®*^ XL (extragrande), con forma de abeja, durante la venopunción. Inicialmente, la enfermera a cargo del procedimiento inspeccionó ambas extremidades superiores del niño o adolescente para seleccionar la vena y el sitio de punción.

A continuación, aproximadamente un minuto antes de realizar la punción, la investigadora fijó las alas de abeja de silicona, almacenadas en el refrigerador de la sala de cardiología pediátrica, al cuerpo vibratorio de *Buzzy* ^*®*^ . El dispositivo se colocó sobre el punto de entrada de la aguja y se fijó con una banda de silicona incluida en el mismo, lo que permitió al profesional tener las manos libres durante el procedimiento. Una vez posicionado y fijado, el dispositivo se encendió de 30 a 60 segundos. En el momento de la punción, el dispositivo se desplazó aproximadamente cinco centímetros por encima del lugar de la punción y permaneció encendido durante al menos otros 60 segundos, tras la finalización del procedimiento.

Para garantizar el rigor en el desarrollo de la investigación, el personal de enfermería de la unidad de cardiología pediátrica que realizó la venopunción recibió formación previa sobre el dispositivo y su funcionamiento, así como capacitación sobre el método de aplicación y el procedimiento paso a paso que debía seguirse durante la recolección de datos. Aspectos como el tiempo de aplicación en el punto de entrada de la aguja, la distancia del dispositivo desde dicho punto y el tiempo que el componente frío debía permanecer fuera del refrigerador se estandarizaron entre los profesionales para evitar implicaciones en los resultados. La investigadora principal (LGBS) estuvo presente durante la venopunción en todas las recolecciones de datos, y prestó asistencia en la aplicación del dispositivo.

En los casos en los que fue necesario un segundo intento para obtener acceso periférico (P1, P9 y P14), el *Buzzy* ^*®*^ se mantuvo en el lugar del primer intento hasta que la enfermera identificó el nuevo sitio de punción. Posteriormente, se siguieron los mismos procedimientos para el uso del *Buzzy* ^*®*^ XL en el nuevo sitio.

En algunos casos, después del procedimiento, fue necesario esperar unos minutos para que los niños o adolescentes experimentaran las sensaciones físicas atenuadas de la venopunción. Transcurrido ese período, se iniciaba la entrevista y se le pedía al niño o adolescente que respondiera algunas preguntas, como: “¿Cómo te fue con *Buzzy* ^*®*^ durante el procedimiento?”, “Si 0 representa “Nada de dolor” y 10 es “Máximo dolor”, ¿cuál fue tu nivel de dolor con el uso de la abejita, de 0 a 10?”, “¿Ya te han hecho una punción? ¿Cuál fue tu nivel de dolor, de 0 a 10, en esa experiencia previa?”. A medida que se respondían estas preguntas, se formulaban otras, como “*¿Qué quieres decir?*” y “Cuéntame más sobre esto”*.* Para profundizar en las respuestas encontradas, todos los niños y adolescentes recibieron información previa sobre la escala numérica del dolor.

Antes y después de cada uso, se realizaba la desinfección del *Buzzy* ^*®*^ , frotando una gasa humedecida con una solución de alcohol al 70%. Las entrevistas se grabaron en audio con un teléfono celular y se realizaron de manera pausada para observar la aparición de cualquier malestar o emoción negativa en los participantes, lo cual no se identificó. Seguidamente, se transcribieron íntegramente para su lectura y análisis.

### Análisis de datos

Los datos cualitativos obtenidos a través de las entrevistas semiestructuradas fueron transcritos y su contenido analizado respetando las etapas del análisis de contenido inductivo: preparación, organización y presentación de resultados^13^.

La fase de preparación comenzó con la transcripción de las respuestas a la entrevista semiestructurada. En esta etapa, no existen reglas sistemáticas para el análisis de datos; sin embargo, las palabras del texto se agrupan en categorías de contenido más pequeñas. Para ello, se realizaron lecturas minuciosas a fin de identificar el significado de las respuestas de los participantes y desglosarlo. En la fase de organización, los datos se codificaron, agruparon y sintetizaron abiertamente, a partir de sus similitudes y diferencias. Finalmente, las categorías se construyeron libremente. Los resultados se reportaron nombrando las categorías y subcategorías de acuerdo con las características del contenido analizado^13^.

Respecto a los datos de caracterización de la muestra, las variables dependientes nominales (sexo, diagnóstico, color de piel, parentesco, escolaridad, estado civil, motivo de hospitalización y procedencia) fueron analizadas y descriptas en distribuciones de frecuencias absolutas (n) y relativas (%), y las variables dependientes continuas (edad e ingresos) fueron reportadas mediante análisis de medidas de tendencia central (media) y dispersión (desviación estándar)^14^.

### Aspectos éticos

El proyecto fue presentado ante y aprobado por los Comités de Ética e Investigación (*Comitês de Ética e Pesquisa*, CEP) con Seres Humanos de las instituciones proponente y coparticipante, bajo expediente n.° 6.132.990 y CAAE n.° 68806623.9.0000.5282. Todas las etapas cumplieron con las directrices establecidas en la Resolución 466/2012 del Consejo Nacional de Salud^15^.

En los procedimientos de recolección de datos, tras identificar a los participantes que cumplían los criterios de selección, se presentó a los responsables el tema y los objetivos de la investigación. Tras la aceptación, el Formulario de Consentimiento Informado fue firmado por el tutor o responsable y el Formulario de Asentimiento por el niño/a o adolescente. La información obtenida mediante este estudio se trató de forma confidencial, garantizándose dicha confidencialidad mediante el uso de un código, donde “P” se refiere a la inicial de la palabra “Participante” y los números respectivos, del 1 al 15, corresponden al orden en que se realizaron las entrevistas, seguido de la edad.

Los participantes recibieron orientación sobre sus derechos, los objetivos de la investigación y el riesgo mínimo de malestar emocional durante la entrevista, dada la vulnerabilidad que experimentaban durante la hospitalización. También se les informó sobre la posibilidad de interrumpir su participación en cualquier momento, sin perjuicio alguno.

Se declara que no existe conflicto de intereses en el uso de *Buzzy* ^*®*^ System en esta investigación.

## Resultados

El estudio incluyó a 15 niños y adolescentes con cardiopatías congénitas. La edad promedio de los participantes fue de 11,1±3,6 años, de los cuales siete (n=7) eran niños entre seis y 11 años (47%) y ocho (n=8) eran adolescentes entre 12 y 17 años (53%). La mayoría eran varones (n=8, 53%) y fueron hospitalizados para tratamiento quirúrgico (n=13, 86%). En cuanto al diagnóstico, predominó la cardiopatía acianótica (n=6, 40%).

A todos los participantes se les realizó una punción con un catéter flexible de diámetro 22 o 24, según la evaluación de la enfermera responsable del procedimiento . Las punciones se realizaron en las extremidades superiores, en la fosa antecubital (n=4), en el dorso de la mano (n=7) o en el antebrazo (n=4). Solo en tres punciones (n=3) se necesitaron dos intentos para obtener el acceso.

De los 15 participantes, 13 informaron menos dolor con el uso de *Buzzy* ^*®*^ y dos calificaron el dolor de punción como de la misma intensidad con el uso del dispositivo.

Tras el análisis de las entrevistas se elaboraron cuatro categorías y una subcategoría, las cuales se presentan a continuación.

### Percepción o no de una reducción del dolor

Al preguntarles sobre su experiencia con el dolor al usar *Buzzy* ^*®*^ , la mayoría de los participantes informaron que, gracias al dispositivo, no pudieron sentir completamente la inserción de la aguja durante la punción, lo que redujo el dolor. Según ellos, esto contribuyó a una experiencia de venopunción menos incómoda, con consecuencias positivas para la venopunción en general, ya que evitó el llanto y los movimientos bruscos de las extremidades durante el procedimiento. Además, expresaron su satisfacción con la disminución de la sensibilidad y la consiguiente reducción del dolor.

*La abejita no me dolió. No creo que vuelva a llorar [...] ¡Me gustó mucho! ¡Le voy a dar un besito! (P1, 9 años).*

*[...] En mi experiencia, sentí que entraba la aguja, pero no sentí el pinchazo por la vibración [...] ayuda mucho que tampoco sientas la aguja y entonces la persona termina fascinada como yo (P4, 13 años).*

*Porque me dejó el brazo así, sin vida, no me ardió; el frío ayuda con la vibración; se siente, pero poco. No me dolió casi nada (P13, 13 años).*

*Fue diferente, ni siquiera saqué la mano cuando ella (la enfermera) me la agarró, porque casi no sentí la aguja; fue rápido y fácil [...] No sentí mucha molestia. Sentí el pequeño aparatito, ¿sabes? Fue bueno porque parece que el dolor disminuyó un poco (P15, 12 años).*

Para los participantes, *Buzzy* ^*®*^ logró reducir significativamente la sensación de dolor del pinchazo de la aguja durante la venopunción en comparación con los procedimientos con agujas realizados antes de la investigación, sin el uso del dispositivo.

*Investigadora: En una escala de 0 a 10, ¿cómo calificarías tu dolor en esas otras experiencias? Adolescente: Creo que fue un ocho o un nueve. Investigadora: Y ahora, después de usar la abejita, en una escala del 0 al 10, ¿cuánto dolor sentiste? Adolescente: Tres (P4, 13 años).*

*Investigadora: Y en una escala de 0 a 10, ¿cuánto dolor sentiste con las otras inyecciones que te pusieron antes? 0 significa nada de dolor y 10 significa mucho dolor. Adolescente: Me dolió mucho. Un ocho. Investigadora: Y con la abejita, en una escala de 0 a 10, ¿cuánto dolor sentiste con la inyección? Adolescente: Solo dos (P6, 15 años).*

*Investigadora: Si tuvieras que poner una nota, en que 0 es “No pica nada”, 1 es “Pica un poco”, 2 quiere decir “Pica un poquito más”, 3 “Pica más”... hasta 10, que es “Pica mucho”, ¿qué nota le pondrías? Niño: Diez. Investigadora: Entiendo, ¿y hoy con la abejita? ¿Qué nota le pondrías? Niño: Uno (1) (P11, 8 años).*

Dos participantes (n=2) reportaron experiencias de dolor equivalentes, en lo que se refiere a la intensidad del dolor, con y sin la abejita; estos fueron los mismos participantes que requirieron dos punciones para el éxito del procedimiento. Según ellos, el dolor experimentado con el uso del dispositivo durante la venopunción fue muy similar al sentido en experiencias pasadas.

*Investigadora: Entonces, en una escala de 0 a 10, ¿cuánto dolor sentiste al utilizar la abejita? Adolescente: Bueno, le daré la misma nota que para sacarme sangre normalmente, alrededor de un 5 o 6 (P9, 13 años).*

*Investigadora: ¿Y qué nota le pondrías, de 0 a 10, al dolor que sentiste cuando te pincharon con la abejita? Niño: Le daría un 7. Investigadora: ¿Y sin la abejita las otras veces? Niño: Un 7 también. Fue muy parecido, me dolió igual (P14, 9 años).*

### **Indicación de posibles mejoras en el uso de *Buzzy*** ^*®*^

Las percepciones de algunos participantes sobre el uso del dispositivo permitieron reconocer algunas limitaciones y sugerir mejoras para el uso de esta herramienta con el fin de potenciar la reducción del dolor. Dos participantes (P5 y P9) destacaron que el componente frío podría ser más efectivo si el tiempo de congelación fuera mayor, y otro (n=1) indicó que la vibración era de baja intensidad y, por lo tanto, apenas se percibía. Cabe destacar que estos dos participantes fueron los mismos que requirieron más de una punción para el éxito del procedimiento, lo por lo que fue necesario más tiempo para realizarlo.

*Si hubiera estado un poco más fría, creo que podría haber anestesiado mejor. No sentí la vibración. [...] Solo sentí sus alas (P9, 13 años).*

*[...] Si la abejita hubiera estado un poquito más congelada... porque estaba como líquida, ¿no? (P5, 11 años).*

### Presencia de sensaciones físicas de relajación y anestesia.

A través de las declaraciones de los participantes, se observó que *Buzzy* ^*®*^ es capaz de proporcionar sensaciones de relajación y anestesia. Para ellos, el hielo y la vibración, en contacto con la piel, provocan sensaciones de relajación muscular, entumecimiento, hormigueo y anestesia en la extremidad punzada. Por lo tanto, sienten que la zona punzada está masajeada, paralizada o incluso entumecida y sin vida. Como consecuencia, la zona de punción se vuelve menos sensible durante el procedimiento, lo que les ayuda a afrontar el temido momento de la punción.

*Es bueno. [...] Porque te relaja. [...] Sientes una relajación que te hace sentir bien (P7, 9 años).*

*Vibraba, ¿verdad? Luego se movía y luego se relajaba un poco (...) El brazo, ¿sabes? Entonces sentí el brazo como si tuviera anestesia, pero solo el brazo. La anestesia es sentir el brazo como si estuviera dormido y no sentir dolor en la zona. Porque el hielo adormece cuando se aplica sobre algo. Como aquí, ¿verdad? (señala la ubicación del Buzzy* ^*®*^ ) (P9, 13 años).

*Ella hizo que no me doliera, sentí un hormigueo y nada más (P10, 6 años) (...) no me dolió nada, por esa sensación de hormigueo y temblor, no se siente la aguja normal (P10, 6 años).*

*Cuando me puso hielo, se durmió para poner la aguja, y ni se sentía la aguja. Fue porque el hielo hizo que se durmiera la mano, y no se sentía la aguja; solo sentí un pinchazo muy pequeño (P12, 13 años).*

*Porque ella lo mueve de esa manera diferente, siento un hormigueo en el brazo en ese momento y se me paraliza un poco. Siento que está menos sensible (P14, 9 años).*

### **Distracción con *Buzzy*** ^*®*^

Los niños y adolescentes también hicieron comentarios sobre la apariencia de *Buzzy* ^*®*^ , usando palabras como bonito, interesante, parecido a un juguete, alegre y feliz. Estos adjetivos se relacionaban con la distracción que proporcionaba durante el procedimiento con la aguja, que suele ser muy estresante. Sus relatos también revelaron el deseo de interactuar con el dispositivo.

Evaluaron la apariencia del instrumento como una ayuda para brindar calma, distracción y felicidad, y relacionaron la forma de abeja con su familiaridad con dibujos presentes en su vida cotidiana. Para este público, todas estas características le dan atractivo al dispositivo y ayudan a entretenerlos y distraerlos de la venopunción.

*Es bonita. Me pareció muy interesante porque su apariencia sin duda tranquilizará al niño; le parecerá linda y la distraerá. Hará que el niño no piense en la aguja sino en la abejita. (P2, 14 años).*

*Porque es linda, su carita, es simpática [...] sonríe, es feliz (P14, 9 años).*

*Es como un juguete (...) Por el ruidito. ¡Y también por las alas! También es bueno por su forma. Tiene estos dibujos. La carita sonriente. [...] Es igual al dibujo que hacemos en el colegio, pero nosotros no logramos hacer una carita así. (...) Feliz y contenta. La hace más bonita y dan ganas de agarrarla. [...] Distrae la mente. [...] Ayuda a no pensar en la aguja (P10, 6 años).*

A pesar de ello, el mismo niño de la declaración anterior manifiesta, en contrapartida, la posibilidad de que la apariencia de *Buzzy* ^*®*^ podría provocar sentimientos contradictorios, ya que las abejas pueden producir picaduras dolorosas.

*Niño: Cuando vibró, pensé que la abejita tenía una aguja, pero luego vi que no la tenía y que dejó de doler. Investigadora: ¿Y por qué pensaste que tenía una aguja? Niño: Porque las abejas tienen esa cosa que pica (P10, 6 años).*

### Reducción de la ansiedad

A través de los informes, se observó que *Buzzy* ^*®*^ reduce la ansiedad asociada a la venopunción por dos razones: los niños y adolescentes esperan una reducción del dolor al usar el dispositivo, y las propias características sensoriales del dispositivo (vibración y frío) promueven la analgesia. Por lo tanto, al centrar su atención en el dispositivo y percibir la reducción del dolor, dejan de pensar en la punción y se sienten más tranquilos y relajados. En sus experiencias, el miedo a la aguja, el nerviosismo y el temor asociados al procedimiento se minimizaron con el uso de *Buzzy* ^*®*^ .

Debido a que redujo la tensión en un momento vulnerable, elogiaron la aplicación del dispositivo como una intervención muy buena y encantadora con el potencial de calmarlos.

*[...] El hecho de que imaginemos que no va a doler tanto también nos tranquiliza mucho, nos quita ese nerviosismo que suele estorbar en esos momentos (P2, 14 años).*

*¡Me encantó! Creo que para quienes tienen mucho miedo a las agujas, como yo, y para quienes tienen aversión a la sangre, también es muy bueno en ese momento (P4, 13 años).*

*Me gustó mucho. [...] Me sentí más tranquila, porque me ayudó a no sentir tanto dolor y a no tener miedo (P5, 11 años).*

*Cuando me pusiste la abeja y la dejaste vibrando, ya me sentía mejor porque me tranquilizó [...] te tranquiliza porque hace que no sientas tanto dolor. Siempre me han dado miedo las agujas, pero... no había otra opción, y sentía como si me pincharan sin parar hasta que sacaban la aguja. Y con la abeja, Me sentí mucho mejor (P12, 13 años).*

*Como me dolió menos en ese momento, el susto fue menor. Cuando me pusieron la aguja, como no la sentí tanto como antes, no tuve esa reacción de susto en ese momento [...] Eso tendría que haber estado las otras veces para ayudar a reducir el susto(P15, 12 años).*

## Discusión

Las percepciones y experiencias subjetivas de los participantes indican que el dispositivo *Buzzy* ^*®*^ reduce el dolor y la ansiedad durante los procedimientos con agujas en niños y adolescentes con cardiopatías congénitas al generar sensaciones de anestesia y hormigueo, promover la relajación muscular y proporcionar distracción.

Si bien la venopunción se requiere comúnmente en unidades de hospitalización para exámenes y tratamientos, es un procedimiento que genera dolor y, por lo tanto, causa miedo, ansiedad y estrés físico y emocional^16^. Todos estos sentimientos y factores estresantes experimentados continuamente pueden contribuir a perjudicar el crecimiento y el desarrollo de niños y adolescentes, ya que afectan el sistema nervioso central, la cognición y el desarrollo motor y emocional. Además, pueden comprometer la adherencia de los niños y adolescentes a la atención médica en el futuro, lo que hace que este tema sea de suma importancia^17^.

A pesar de los numerosos avances en la evaluación y el tratamiento del dolor en la población pediátrica, aún existe dificultad para cuantificarlo debido a su naturaleza subjetiva, así como para tratarlo adecuadamente. Como consecuencia, los niños y adolescentes con cardiopatías experimentan sufrimiento emocional y sensorial, con riesgo de sufrir cambios hemodinámicos significativos y variaciones en la oxigenación tisular debido a fluctuaciones en la presión intravascular^18^. Por lo tanto, es necesario encontrar una manera de aliviar la experiencia del dolor físico y emocional, incluso durante los procedimientos con agujas, con el objetivo de humanizar la hospitalización y minimizar los impactos negativos en la calidad de vida^19^.

Una revisión reciente de la literatura^18^ investigó las principales intervenciones no farmacológicas para el manejo del dolor en niños mayores de un año sometidos a procedimientos con agujas y concluyó que los tres métodos principales adoptados para la punción venosa periférica fueron el uso de goma de mascar, distracción y *Buzzy* ^*®*^ .

A partir de la Teoría del Manejo de Síntomas (TMS), marco teórico que guió este estudio^7^, se entiende que las respuestas a síntomas como el dolor pueden abarcar las dimensiones fisiológicas, psicológicas y conductuales, y la mejor manera de comprender esta experiencia es a través del autoinforme del individuo que lo experimenta. Por lo tanto, se buscó un enfoque más preciso y profundo de este fenómeno desde una perspectiva cualitativa, centrándose en niños y adolescentes con cardiopatías congénitas, para explorar los significados que atribuyen a las evaluaciones del uso de este dispositivo y cómo responden al síntoma tras la aplicación de la intervención propuesta.

En los informes, se observó que la intervención resultó en una disminución del dolor en casi todos los participantes, excepto en dos, quienes lo percibieron sin cambios. Esto se explica por la relación entre los mecanismos del efecto analgésico local generado durante la punción y la compartición de sinapsis en la médula espinal, donde las fibras que conducen el estímulo doloroso comparten las vías con las fibras de conducción térmica y mecánica. Esta interferencia interneural en la conducción sináptica es capaz de generar la sensación de relajación mencionada por los participantes y, en consecuencia, el alivio del dolor^20^.

La modulación del dolor con *Buzzy* ^*®*^ se produce mediante la influencia del dispositivo tecnológico en el sistema inhibitorio descendente, originado en la sustancia gris del mesencéfalo. En este caso, la información dolorosa que viajaría desde el cerebro hacia el asta dorsal de la médula espinal se reduce debido a cambios serotoninérgicos^21^. Como resultado, el dolor físico y el sufrimiento emocional se alivian con *Buzzy* ^*®*^ .

Además de los beneficios para los pacientes, el procedimiento resulta menos traumático para el profesional que realiza la punción, ya que puede realizarse con mayor rapidez y calma, con menos riesgo de llanto, agitación y movimientos bruscos. Por lo tanto , el uso de *Buzzy* ^*®*^ puede hacer que el procedimiento sea más seguro y aumentar las probabilidades de éxito del profesional en el primer intento de punción. Las sensaciones atribuidas al dispositivo incluyen “no llorar”, “calmarse”, “estar menos nervioso” y “estar más relajado” y contribuyen en este proceso.

Además, la reducción del dolor se acompañó, según los informes, de sensaciones de “anestesia”, “relajación”, “entumecimiento” o “dormición”, “parálisis” y “hormigueo”. La combinación de crioterapia con vibración también potencia su eficacia gracias a la liberación de opioides endógenos resultantes de la activación de los receptores táctiles relacionados con el frío, lo que provoca una sensación de entumecimiento local, con la consiguiente “anestesia”, según informaron los participantes^21^.

Un estudio clínico realizado por la desarrolladora de la tecnología comparó diferentes frecuencias de vibración para determinar cuál se aplicaría mejor al dispositivo. Se descubrió que la frecuencia de entre 180 y 200 Hz aplicada a la tecnología Oscillice de *Buzzy* ^*®*^ es lo suficientemente potente como para superar los efectos de las terapias basadas en estimulación eléctrica en la reducción del dolor posquirúrgico, el dolor espinal y otras lesiones^22^. En concordancia con estos resultados, otro estudio demostró que la vibración mecánica de alta frecuencia proporciona un alivio significativo del dolor musculoesquelético, demostrando ser más eficaz que los métodos tradicionales, incluidos los farmacológicos, lo que corrobora los informes de los participantes en este estudio^23^.

En cuanto a las sensaciones reportadas por niños y adolescentes, es importante destacar que el enfriamiento local obtenido con las alitas de hielo proporciona analgesia y contribuye al entumecimiento al disminuir la tasa metabólica local y reducir el flujo de mediadores inflamatorios. La interrupción de la transmisión de la señal de dolor a través de esta vía aumenta sucesivamente el umbral absoluto del dolor con el aumento de betaendorfinas y, en poco tiempo, los músculos se “relajan” y “adormecen”, lo que facilita el procedimiento de punción^24^. Además de los elementos previamente discutidos sobre el dispositivo que genera analgesia, la distracción es uno de los mecanismos que confieren eficacia a *Buzzy* ^*®*^ y ha demostrado ser eficaz en varios ensayos clínicos aleatorizados^21^.

El aspecto lúdico del dispositivo se refiere al escenario de juego del niño y está estrechamente vinculado con el desarrollo emocional y cognitivo. Según un estudio, cuando el juego es utilizado correctamente por el profesional mediante la atención participativa, este puede ayudar al niño a estructurar los eventos que le rodean, reduciendo la ansiedad, aumentando la aceptación del tratamiento, mejorando la comunicación y contribuyendo a la resignificación del momento de la hospitalización^25^.

Artículos internacionales han identificado que, ante la ansiedad durante procedimientos con agujas, niños y adolescentes pueden presentar un aumento considerable de la frecuencia cardíaca^21^ ^,^ ^26^ ^-^ ^28^, además de hipoxemia y cambios vasovagales y hormonales^21^ ^,^ ^28^. Con el uso de *Buzzy* ^*®*^ , se observó una reducción de los parámetros cardíacos a niveles cercanos a la normalidad^26^ ^-^ ^27^.

Si bien los niños y adolescentes reconocen a *Buzzy* ^*®*^ como una herramienta útil para reducir el dolor durante los procedimientos, los hallazgos revelan aspectos del dispositivo que representan desafíos y posibilidades de mejora. En este sentido, se considera importante desarrollar protocolos más adaptativos que consideren variables de desarrollo, sensoriales y contextuales para optimizar la experiencia de estas personas^26^.

En este estudio, dos participantes no reportaron alivio de la sensación dolorosa. Si bien la mayoría de los estudios demuestran la efectividad del dispositivo, otras investigaciones corroboran la necesidad de estrategias individualizadas^29^. Se destaca que ambos participantes fueron los mismos que no lograron éxito en el acceso periférico en el primer intento. Además , uno de ellos mencionó la oportunidad de mejora con respecto al tiempo de congelación de las alitas. Según un estudio^25^, los intentos repetidos de cateterización periférica pueden aumentar la sensibilidad de niños y adolescentes a este tipo de procedimiento y potenciar el dolor, ya que los procedimientos con agujas son la principal causa de temor en estos pacientes^25^.

Otro participante que requirió una segunda punción reportó alivio del dolor durante el procedimiento. Por lo tanto, se cree que varios factores pueden influir en la sensación dolorosa, como la etapa de desarrollo del niño^7^ ^,^ ^28^, la mayor dificultad para modular el dolor en niños menores de siete años, las variaciones en la aplicación de la técnica, el tiempo de exposición a la crioterapia, las preferencias individuales respecto a colores y formas que podrían no coincidir con la presentación del dispositivo^24^ ^,^ ^26^ ^)^ y la presencia de los padres^30^.

Contrariamente al diseño lúdico del dispositivo, hubo una asociación entre la forma de abeja de *Buzzy* ^*®*^ con riesgo inminente de una sensación dolorosa relacionada con la picadura del insecto. Los niños más pequeños aún incorporan elementos fantásticos a sus realidades^20^, lo que puede hacer que la abejita resulte aterradora para algunos. En este caso, optar por la variante de la mariquita sería una opción.

Además, dos participantes consideraron que el tiempo de congelación de las alas de la abejita era insuficiente para proporcionar una analgesia adecuada, ya que, durante la punción, ya estaban en estado líquido. Se sabe que el tiempo de descongelación del componente frío, desde el momento en que se saca del congelador, es de 10 minutos^23^. Esto significa que, para procedimientos más largos o con mayores retrasos operativos, sería importante retirar el ala del refrigerador lo más cerca posible del inicio o sustituirla por otra.

Las limitaciones del estudio incluyen la necesidad de un diseño de *Buzzy* ^*®*^ más adecuado según el rango de edad de los usuarios, así como el reemplazo de las alas de silicona congeladas cuando se dejan fuera del refrigerador por más de 10 minutos.

Dar voz a niños y adolescentes es una importante estrategia de empoderamiento e inclusión en el proceso de atención, ya que facilita el acercamiento de estos pacientes al equipo de salud y al conocimiento científico. Paralelamente, estudios refuerzan que el relato de niños y adolescentes sobre su situación de salud o sus experiencias es la mejor manera de comprender sus perspectivas, lo que hace que cada declaración sea sumamente valiosa para la comprensión de la totalidad^8^ ^,^ ^10^ ^,^ ^25^.

Como fortaleza de este estudio, es posible destacar el estímulo al uso de intervenciones no farmacológicas por parte del equipo de salud, algo que aún es escaso en la práctica clínica. Esto se puede observar, por ejemplo, en el estudio que evaluó 1.251 historias clínicas de niños hospitalizados e identificó, en solo seis de ellos, notas de enfermería sobre el uso de medidas no farmacológicas para el manejo del dolor, como calor local, distracción y compresas frías^2^. Se entiende que el TMS, al estar compuesto por tres componentes (la experiencia del síntoma - en este estudio, dolor; estrategias para el manejo de los síntomas (*Buzzy* ^*®*^ ); y resultados - dolor con el uso de *Buzzy* ^*®*^ ), tiene el potencial de guiar intervenciones de enfermería dirigidas al manejo adecuado de los síntomas en la práctica, influir en las prácticas institucionales y crear entornos de atención propicios para mejorar la calidad de vida de esta población^8^.

Es necesario realizar más investigaciones sobre el uso de *Buzzy* ^*®*^ en niños y adolescentes sometidos a procedimientos dolorosos con agujas para profundizar en estos hallazgos e investigar la experiencia de esta población pediátrica en una mayor variedad de procedimientos, tratamientos (hospitalarios y extrahospitalarios) y diagnósticos, y bajo diferentes diseños (como ensayos clínicos aleatorizados o enfoques mixtos). Esto permitirá una comprensión más profunda de los efectos de *Buzzy* ^*®*^ .

## Conclusión

Las cuatro categorías encontradas (“Percepción o no de una reducción del dolor”; “Presencia de sensaciones físicas de relajación y anestesia”; “Distracción con *Buzzy* ^*®*^ ”; y “Reducción de la ansiedad”) resaltan la experiencia de niños y adolescentes con enfermedades cardíacas al utilizar *Buzzy* ^*®*^ durante la venopunción. Según los participantes, se trata de una intervención no farmacológica eficaz, interesante y placentera que actúa tanto en su dimensión física como emocional, ya que experimentan menos dolor, ansiedad y nerviosismo durante el procedimiento, con mayor relajación y distracción.

Se cree que los resultados de este estudio pueden contribuir a futuras investigaciones, puesto que implican una reducción de las lagunas nacionales e internacionales identificadas durante las búsquedas bibliográficas. Además, contribuyen a concientizar a otros profesionales sobre la importancia de utilizar estrategias para reducir el dolor durante la venopunción, con el fin de mejorar la atención de enfermería que se brinda a niños y adolescentes y hacer que la atención sea más humana y menos traumática.

## Data Availability

Los conjuntos de datos relacionados con este artículo estarán disponibles previa solicitud al autor correspondiente.
